# Pathology Competencies in Medical Education and Educational Cases: Update 2023

**DOI:** 10.1016/j.acpath.2023.100086

**Published:** 2023-07-12

**Authors:** Barbara E.C. Knollmann-Ritschel, Alison R. Huppmann, Michael J. Borowitz, Richard Conran

**Affiliations:** a Department of Pathology, Uniformed Services University of the Health Sciences, Bethesda, MD, USA; b Departments of Biomedical Sciences and Pathology, University of South Carolina School of Medicine Greenville, Greenville, SC, USA; c Department of Pathology, Johns Hopkins Medical Institutions, Baltimore, MD, USA; d Department of Pathology and Anatomy, Eastern Virginia Medical School, Norfolk, VA, USA

**Keywords:** Pathology competencies, Pathology objectives, Educational cases, Disease mechanisms, Organ system pathology, Diagnostic medicine, Therapeutic pathology

## Abstract

Pathology is a core component of medical school curricula because understanding the pathogenesis of the disease is foundational both for diagnostic efficiency and optimal use of ancillary resources in patient care. The Pathology Competencies for Medical Education (PCME) were developed as a national resource of expectations of pathology knowledge for medical students. The PCME are composed of three competencies: disease mechanisms and processes, organ system pathology, and diagnostic pathology and therapeutic pathology. The learning goals and learning objectives of the PCME that were first published in 2017 have been carefully revised and updated. Significant additions were made to fill gaps of the original PCME objectives, and some learning objectives have been retired or moved to more appropriate locations within the competencies. As curricula and the practice of medicine change, the PCME will continue to be revised and updated periodically. They have and will continue to serve as the organizing principle for the growing number of educational cases published by *Academic Pathology*. Nomenclature in the original and revised PCME will allow for continued linking of previous and new educational cases to the revised learning objectives. PCME and the educational cases can be adapted into any type of curricula. Having a widely accepted resource of learning objectives in pathology will help students and medical educators focus on essential components of pathology for the future practice of medicine.

## Introduction

A great shift in medical school curricula has occurred in the past 10 to 15 years from course-based curricula to integrated organ system-based curricula. This shift has allowed medical students to enter clerkships earlier and increase their clinical exposure. However, practicing medicine today, perhaps more than ever, requires a broad knowledge of normal and pathological processes. The rapid increase in the number of available laboratory tests, most notable in the explosion of genetic testing, makes it even more important for practicing physicians to have a solid understanding of mechanisms of disease, both to choose the most appropriate laboratory test(s) and to counsel their patients about their meaning. This project began approximately 10 years ago as a working group of the Association of Pathology Chairs (APC), which developed an initial set of pathology competencies described in “National Standards in Pathology Education."^1^ In this initial publication, over 60 pathology course directors and pathology chairs submitted course objectives that were extensively reviewed and, in 2014, posted on the APC website. To further disseminate these learning objectives, the APC published a revision of them in *Academic Pathology* in July 2017 as the Pathology Competencies for Medical Education (PCME).^2^ The objectives are placed within one of three competencies: (1) Disease Mechanisms and Processes, (2) Organ System Pathology, and (3) Diagnostic Pathology and Therapeutic Pathology.

Just prior to its annual meeting in 2018, the APC sent a survey to its members to evaluate the impact of the PCME and to assess the extent to which these had been implemented in curricula. Forty-nine members from 48 different institutions responded to the survey. Of the responders, 42 (86%) responders indicated that they had read the PCME and 29 (59%) responders indicated that they had used information from the PCME to change, modify, or update the learning objectives at their own institution. Comments received indicated that members had altered lectures to align with the PCME; updated course content and/or learning objectives; mapped their own institution curriculum on to the PCME. Others found out about the PCME from the survey and indicated that they now plan to use them. Some responders commented that the PCME should be vetted nationally with other national society buy-in, and a rare comment mentioned that the objectives were too dense. Sixteen (34%) of responders indicated that the PMCE were useful in other ways, such as to negotiate for more time with institutional leadership, to develop 4th year electives, to map curriculum, to re-affirm what pathology course directors are teaching, and to add diagnostic medicine to the curriculum. There were also some suggestions for additions to fill gaps in objectives, including interstitial lung disease, head and neck (HN) pathology, multisystem disease and amyloidosis, urinalysis, and chemistry (CHEM). This current revision is an attempt to address the previous comments and the gaps in the PCME and to provide a more uniform and updated set of competencies for educators to use in undergraduate medical education (UME).

## Methods

**Fig. 1:**
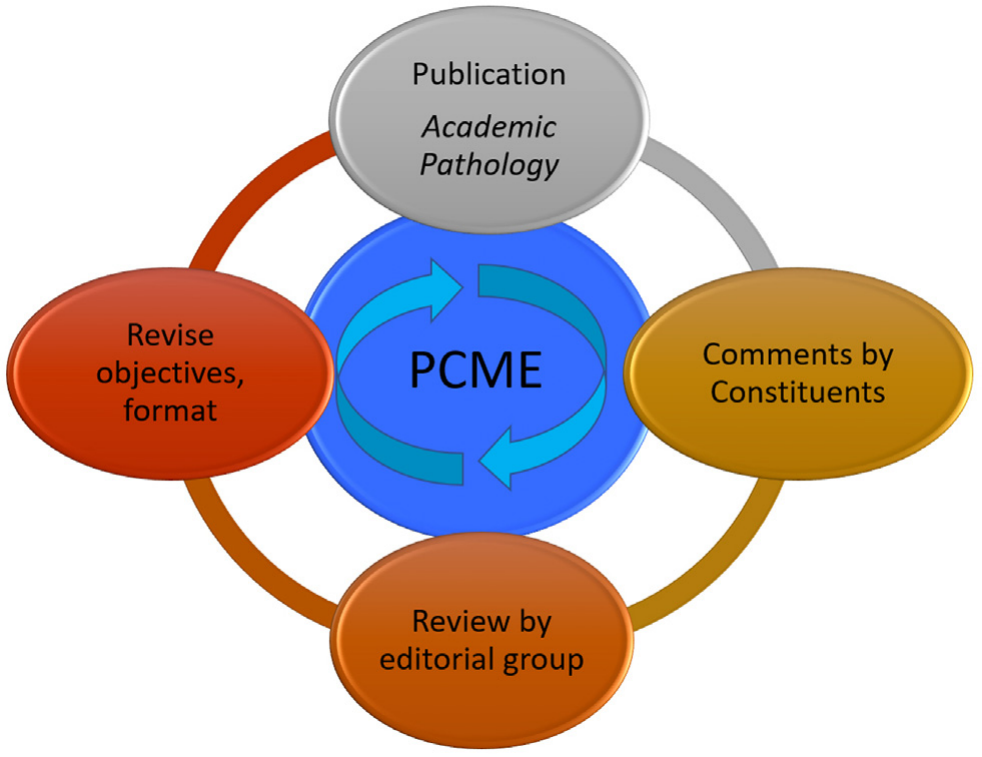
Review cycle of the Pathology Competencies in Medical Education. To ensure the learning objectives stay current with the practice of medicine, objectives are reviewed periodically to incorporate comments by constituents, reviewed by an editorial group, and published as a revised version.

Since the first publication of the PCME in 2017, there have been many opportunities for comments of the learning objectives. In addition to the formal survey noted above, faculty have provided direct input to the authors of the PCME and sessions have been devoted to the PCME at conferences. Many authors who have submitted educational cases have commented on the PCME; in turn, the editors of the educational cases identified gaps when reviewing submitted cases. We have taken the comments from these many sources and have systematically reviewed, reorganized, and revised the PCME. In order to decide whether an objective merited inclusion we used standard pathology textbooks and a recent comprehensive reference to assess whether information was necessary to include.^3^, ^4^, ^5^ As Fig. 1 illustrates, the revision of the PCME is a cyclic process that includes incorporation of comments from constituents through multiple processes mentioned above.

We have removed redundancies and made some significant shifts in several topic areas so that the learning objectives are more appropriately aligned with the 3 major competency areas. We also added significant amounts of new content. For example, in genomics, several mechanistic objectives in competency 3 were moved to competency 1. In hematopathology, pathophysiologic objectives formerly in competency 3 were moved to competency 2; diagnostic objectives are now collected in competency 3. To fill gaps, we sometimes introduced brand new learning goals, such as those representing vascular disorders, multisystem disease, or HN pathology; other gaps were filled by adding objectives within an existing learning goal. Competency 3 was significantly revised to expand the CHEM and the transfusion sections; learning objectives throughout this competency are now focused more specifically on diagnostic testing. Finally, several learning objectives have been retired because they either did not fit with current pathology teaching or duplicated other learning objectives.

The nomenclature of the original learning objectives has been retained, and notes have included to indicate any change from the prior PCME edition. If a learning objective was moved to a different competency, we have used the nomenclature “C” for competency along with the prior learning goal and objective to indicate its origin. For example:

“Objective GM1.7 (Formerly C3, GE1.4): Linkage Analysis. Outline the principles that underlie genetic linkage analysis and association studies and how they are used to identify genes associated with diseases.”

This learning objective of linkage analysis is now objective GM1.7 in competency 1 under genetic mechanisms (GMs), and was moved from competency 3, GE 1.4, genomics.

We hope this nomenclature will allow continued easy cross reference for both prior and current learning objectives to allow for consistent mapping of any previously published educational case attached to an earlier learning objective. Publication in *Academic Pathology* ensures wide access to the revised learning objectives.

## Discussion

The PCME were originally designed to represent the minimum knowledge thought necessary for medical students to practice in medicine today. Through the revisions of the PCME, we have retained the three major competencies under which the learning objectives are now better aligned. These objectives are broad and comprehensive and can be applied to different curricula, whether in a free-standing course or adapted into the current model of integrated teaching. While relevant to a comprehensive pathology curriculum, we acknowledge that not all learning objectives need to be taught by pathologists. We hope that having peer-reviewed learning objectives as a national resource will encourage pathology educators to adapt some or all into their curricula or to refine their own learning objectives. As confirmation that a foundational understanding of pathology continues to be central to UME, approximately 50% of the questions in the United States Medical Licensing Exam (USMLE) Step 1 assess pathology material.^6^

In addition to integrated curricula, some medical schools have shifted to competency-based medical education by endorsing Entrustable Professional Activities (EPAs). Several of the EPAs developed by the Association of American Medical Colleges tie directly to the foundational understanding of pathology. These include EPA 3 “Recommend and Interpret common diagnostic and screening tests”; EPA 2 “Prioritize a differential diagnosis following a clinical encounter”; EPA 9 “Collaborate as part of an interprofessional team.“.^7^ In addition, the third pillar of medical education, Health System Science (HSS),^8^ requires an understanding of the important role pathology plays to ensure safe, equitable, timely, and efficient patient care. This will lead to an increase in efficient use of laboratory testing and an increase of patient safety. Publications integrating HSS curriculum in UME include a 2016 publication from the American Medical Association describing the domains of HSS education.^9^

While the learning objectives are designed as free-standing statements to capture the breadth and depth of necessary content in pathology, one of their major uses has been as an anchor for the development of educational cases. These are succinct peer-reviewed clinical case presentations that lead the reader through clinical reasoning to determine a differential diagnosis based on history, physical examination, and laboratory findings. The educational cases all have a primary learning objective and may have additional secondary learning objectives. An educational case focused on basic pathobiology would typically have a primary C1 learning objective, one focused on organ system pathology C2, and one on laboratory diagnosis of disease C3. Most cases have multiple objectives; the primary learning objective should be fully discussed in the educational case, but secondary learning objectives do not need full discussion. Teaching points at the end of each case discussion provide the key learning points of the primary learning objective and secondary objectives if used.

The repository of educational cases that accompany the PCME continues to grow by approximately 40 new cases per year. Currently, 169 have been published in *Academic Pathology*; additional cases are in production. Educational cases comprise 4 of the top 10 articles downloaded from *Academic Pathology* in 2022. In addition, 9 of the top 10 accessed publications from *Academic Pathology* in PubMed Central® are educational cases. We encourage authors to continue to submit educational cases, especially in the areas of new and revised learning objectives. Cases can easily be adapted into many different types of learning settings, including laboratories, small group discussions, and team-based learning exercises. The cases emphasize the use of clinical reasoning to devise a differential diagnosis and provide increasing amounts of information to the learner to refine a differential and arrive at a final diagnosis. The discussions that accompany the cases emphasize the clinical reasoning behind the conclusions and should fully explain all aspects of the primary learning objectives. By incorporating clinical, laboratory, and pathophysiologic information, these exercises help undergraduate medical students develop the critical thinking skills they will need as practicing physicians. With the nomenclature system developed for the PCME, educators can search for cases meeting specific objectives using the spreadsheet on the APC website.^10^ The linkage between previous versions and this revision will allow them to find cases written under a previous leaning objective that was moved or retired.

While medical educators are the primary audience of the PCME and the educational cases, students can also use the cases for study, review, and expansion of knowledge. While not designed to reflect what every student must master before beginning a residency, the PCME can serve as a checklist for students to follow as they learn the breadth and depth of pathology; the educational cases provide vehicles for them to see how that knowledge is applied.

The most extensive changes in this revision are to the third competency on diagnostic medicine. A previous report indicated that nearly all inpatient care, just over half of emergency room visits, and about one third of outpatient visits result in laboratory testing,^11^ underscoring the need for all medical students to understand laboratory medicine. We now include many new learning objectives in this competency, and its focus is now clearly to equip the medical student with a broad understanding of laboratory testing. Knowing when and how to order tests (including appropriate diagnostic use of tissue samples), what information they provide, and how disease processes produce the abnormalities detected all will help future physicians provide cost effective and efficient patient care. A recent publication highlighting the most common laboratory tests and the pathophysiology underlying changes in their values has been linked to the PCME.^5^

## Conclusion

The revised PCME highlight three major competencies in pathology, namely disease mechanisms and processes, organ system pathology, and diagnostic medicine and therapeutic pathology. The revisions have addressed gaps in the original PCME publication and reorganized and revised existing objectives to better align with the competencies. The nomenclature system was retained from the original PCME publication and was expanded to allow easy cross reference when learning objectives were moved and combined. The nomenclature system should allow for the continued referencing of educational cases to the pathology competencies. Knowledge of pathophysiology and laboratory diagnosis continues to be essential for the practice of medicine. The PCME serve as a national peer-reviewed resource of learning objectives that should be beneficial for both students and medical educators. However, the document is designed to be a living document that is periodically revised and updated to ensure relevance as medical practice evolves. We continue to encourage comments about the pathology competencies to be sent to the authors. We also encourage continued submission of educational cases to *Academic Pathology* to grow this international resource.

## Competency 1

### Disease Mechanisms and Processes

A foundational knowledge of adaptive changes and mechanisms of disease including the etiology, local or systemic responses, effects, pathogenesis, consequences, molecular and cellular events is essential for understanding disease processes in organ system pathology and in patients.

There are 12 topics within this competency area. Each topic includes general learning goals and specific objectives that students should be able to meet before step 1 of the USMLE. Table 1 lists the topic areas and reference codes and shows the number of goals and objectives for each.

**Table 1:** Disease mechanisms and processes.

Topic	Number of Goals	Number of Objectives	Reference Code
Genetic mechanisms	1	12	GM
Neoplasia	3	12	N
Environmental mechanisms	2	9	EM
Metabolic and nutritional mechanisms	1	5	MN
Inflammatory mechanisms	1	8	FLAM
Immunological mechanisms	1	11	IM
Infectious mechanisms	2	13	FECT
Tissue renewal, regeneration, and repair	1	7	RRR
Hemodynamic disorders and thromboembolic disease	2	8	HDTD
Adaptation and cell death	3	7	ACD
Developmental processes	1	2	DEV
Geriatrics	1	1	GER

### Topic: Genetic Mechanisms (GM)

This topic includes a basic knowledge of genetic mechanisms of disease, including inherited and somatic disorders, with the resulting consequences leading to disorders of development, metabolism, aging, stem cell biology, immunology, and the development of cancer.

#### Learning Goal 1: Genetic Mechanisms of Developmental and Functional Abnormalities

Apply knowledge of the genetic mechanisms of disease to discuss how changes in the genome can cause developmental and functional abnormalities at the cellular, tissue, and organism levels.  

*Objective GM1.1*: *Types of Mutations*. Describe different types of mutations that can occur in human disease and discuss how each of these can produce abnormalities in DNA transcription and/or alterations in the type or amount of protein produced.  

*Objective GM1.2: Inheritance Patterns*. Compare and contrast the inheritance patterns of different types of Mendelian disorders and give examples of each type of pattern.  

*Objective GM1.3: Genetic Diseases of Enzyme Function*. Provide examples of genetic diseases associated with abnormal enzyme function and compare and contrast with genetic diseases that produce other abnormal proteins.  

*Objective GM1.4: Chromosomal Abnormalities*. Discuss mechanisms that result in developmental abnormalities involving abnormal chromosomal number and provide examples of diseases associated with trisomies or chromosomal deletions.  

*Objective GM1.5: Multifactorial Inheritance and Environmental Factors*. Discuss and give examples of disorders associated with multifactorial inheritance and describe how environmental factors can interact with genetic factors to produce or modulate disease.  

*Objective GM1.6: Nonclassical Inheritance or Mitochondrial Inheritance*. Describe the pathophysiologic mechanisms that result in disorders of a nonclassical inheritance and mitochondrial inheritance and give clinical examples of each.  

*Objective GM1.7 (Formerly C3, GE1.4): Linkage Analysis*. Outline the principles that underlie genetic linkage analysis and association studies and how they are used to identify genes associated with diseases.  

*Objective GM1.8 (Formerly C3 GE1.5): Population Genetics*. Define the concepts “founder effect” and “genetic drift” and explain how genetic variants are distributed within populations.  

*Objective GM1.9 (Formerly C3 GE1.6): Genetic Risk*. Explain how genetic risk is determined by carrier status and carrier frequency of a condition and determine carrier frequencies and incidence of recessive conditions using the Hardy-Weinberg Law.  

*Objective GM1.10 (Formerly C3 GE1.7): Phenotypic Expression*. Distinguish dominant and recessive phenotypes and alleles and describe how incomplete penetrance, variable expressivity, imprinting, and pleiotropy affect the phenotypic expression of diseases.  

*Objective GM1.11 (Formerly C3 GE1.8): Modifier Genes*. Describe the concept of a modifier gene and its contribution to phenotypic variability.  

*Objective GM1.12 (Formerly C3 GE1.10): Mosaicism*. Define mosaicism and explain how it affects the phenotype of a chromosomal disorder.

### Topic: Neoplasia (N)

This topic includes a basic understanding of the characteristics of benign and malignant neoplasms, epidemiologic, and environmental factors that influence neoplastic change, as well as an understanding of the molecular basis of neoplasia including oncogenes, tumor suppressor genes, carcinogenic agents, and host defense.

#### Learning Goal 1: Genetic Basis of Neoplasia

Apply knowledge of the genetic basis of neoplasia to explain how genetic changes are acquired, how functional alterations in these mutated genes lead to the development of cancer, and how these alterations can be exploited with therapy.  

*Objective N1.1: Genetic Mechanisms of Neoplasia*. Discuss and provide examples of molecular genetic mechanisms that underlie cancers, including germline mutations, somatic mutations (including point mutations, deletions, amplifications, and translocations), epigenetic changes, and DNA repair gene effects.  

*Objective N1.2: Oncogenes and Tumor Suppressor Genes*. Explain the action of oncogenes and tumor suppressor genes in growth factor-initiated signal transduction in both normal and neoplastic cells and discuss how this information can be utilized for treatment.  

*Objective N1.3: Genes that Promote Growth or Inhibit Cell Death*. Compare and contrast the actions of genes that promote cell growth in cancers with those that inhibit cell death and explain how this information influences the choice of therapeutic agents.  

*Objective N1.4: DNA Fidelity*. Describe how cells maintain DNA fidelity and discuss, with examples, how mutations in these pathways produce genomic instability and clonal evolution.

#### Learning Goal 2: Environmental Influences on Neoplasia

Apply knowledge of the environmental factors that influence neoplastic transformation.  

*Objective N2.1: Environmental and Geographic Impact on Neoplasia*. Describe how environmental factors influence the prevalence of neoplastic diseases and discuss how this influence alters as patients move between geographical regions.  

*Objective N2.2: Mechanisms of DNA Damage Repair*. Describe the mechanisms by which exposure to radiation, tobacco, alcohol, or other environmental chemical agents can produce cancer.  

*Objective N2.3: Influence of Viruses or Microbial Agents on Neoplasia*. Describe the mechanisms by which viruses and other microbiological agents can contribute to the development of cancer.  

*Objective N2.4: Environmental Factors that Influence Neoplasia*. Retired.  

#### Learning Goal 3: Characteristics of Neoplasia

Apply knowledge of the characteristics of neoplasia to discuss the pathogenesis, morphologic appearance, classification, biological behavior, staging of neoplasms, and mechanisms of paraneoplastic syndromes.  

*Objective N3.1: Morphologic Features of Neoplasia*. Describe the essential morphologic features of neoplasms and indicate how these can be used to diagnose, classify, and predict biological behavior of cancers.  

*Objective N3.2: Cellular Capabilities of Neoplasia*. Discuss the cellular capabilities of neoplasms that enable them to invade tissues and to metastasize and recognize how this differentiates benign from malignant neoplasms.  

*Objective N3.3: Stromal Elements in Cancer*. Discuss the dependence of cancers on stromal elements including blood supply and explain how this information can be used to treat cancers.  

*Objective N3.4: Paraneoplastic Syndromes*. Define and provide examples of paraneoplastic syndromes and describe how substances produced by cancers can produce systemic effects in the host.  

*Objective N3.5: Grading and Staging of Neoplasia*. Compare and contrast the basic grading and staging of neoplastic diseases and describe the tumor, (lymph) nodes, and metastasis classification for common tumors such as breast and colon carcinoma.

### Topic: Environmental Mechanisms (EM)

This topic includes etiologies such as physical damage resulting from trauma, particles, extreme temperature, radiation, and chemical exposures to small molecules and biologic toxins that produce tissue damage. The mechanism of injury usually causes direct damage that initiates a host response that can lead to a range of consequences from resolution to a chronic complicated pathologic state.

#### Learning Goal 1: Injury from External Agents

Apply knowledge of biochemistry and cellular physiology to describe the mechanisms leading to cell or tissue injury induced by exposure to external agents, including radiation, environmental toxins, drugs of abuse, and therapeutic agents.  

*Objective EM1.1: Mechanisms of Cell Injury*. Compare and contrast different mechanisms of chemical injury, specifically agents that act by direct binding to and inactivation of cellular constituents with those that require metabolic activation to induce toxicity.  

*Objective EM1.2: Tobacco Use*. Discuss the pathogenesis of tobacco use and the resultant pathologic changes in affected organs.  

*Objective EM1.3: Alcohol Use*. Discuss the pathogenesis of alcohol use and the resultant pathologic changes in affected organs.  

*Objective EM1.4: Substance Use Disorders*. Describe the mechanism by which inappropriate use of substances induces central nervous system effects and discuss, with examples, toxicities associated with both chronic use and acute overdose of these drugs as well as withdrawal effects.  

*Objective EM1.5: Occupational Exposure*. Provide examples of industrial, occupational, or environmental exposures that produce disease and the resultant pathologic changes in these affected organs from chronic exposure and indicate what organ systems are most commonly affected by which agents.  

*Objective EM1.6: Toxicity of Therapeutic Drugs*. Discuss, with examples, how therapeutic drugs can produce toxic effects on different tissues, distinguishing between idiosyncratic and dose-dependent effects.  

*Objective EM1.7: Radiation*. Discuss the mechanisms by which radiation damages cells and tissues and compare and contrast how ultraviolet radiation, therapeutic radiation, and acute radiation sickness produce different disease manifestations in different organ systems.

#### Learning Goal 2: Physical Injury

Apply knowledge of biochemistry, anatomy, physiology, and mechanisms of cell injury to describe the pathogenic mechanisms of physical injury.  

*Objective EM2.1: Mechanical Force Injury*. Compare and contrast the types of injuries associated with mechanical force (blunt versus penetrating) with respect to effects on skin, blood vessels, and the affected organs and discuss systemic response to massive trauma.  

*Objective EM2.2: Thermal Injury*. Discuss thermal injuries, comparing and contrasting the direct and systemic effects of thermal burns, hyperthermia, and hypothermia and mechanisms of injury at the cellular level.

### Topic: Metabolic and Nutritional Mechanisms (MN)

This topic includes the etiologic mechanisms, host responses, and disease processes leading to impairment of absorption, transport, and utilization of nutrients and oxygen, storage disorders, and disposal of waste products.

#### Learning Goal 1: Nutrient Deprivation or Toxicity

Apply knowledge of biochemistry and cellular physiology to explain the pathogenic mechanisms resulting from nutrient deprivation or toxicity and the resulting pathology at the cellular, tissue, and organism levels.  

*Objective MN1.1: Fat- and Water-Soluble Vitamins*. Compare and contrast dietary sources of fat-soluble and water-soluble vitamins with respect to absorption, metabolism, and potential toxicity.  

*Objective MN1.2: Vitamin Deficiency Disorders*. List vitamins and minerals whose deficiency can be associated with a disease process and explain the mechanisms by which these deficiencies produce disease.  

*Objective MN1.3: Obesity*. Discuss the etiology, pathogenesis, and common clinical consequences of obesity, comparing and contrasting genetic and environmental factors.  

*Objective MN1.4: Malnutrition*. Discuss the pathologic mechanisms and consequences of nutritional deficiencies other than vitamin deficiencies, with emphasis on severe protein-energy malnutrition, and discuss the pathologic states that have a significant impact on nutritional requirements.  

*Objective MN1.5: Diet and Systemic Disease*. Discuss the effect of diet and nutritional state on systemic disease, emphasizing the role it plays in the development of cardiovascular disease and cancer.

### Topic: Inflammatory Mechanisms (FLAM)

This topic includes the understanding of acute and chronic inflammation, including patterns of inflammation, cellular components, mediators, and systemic effects.  

#### Learning Goal 1: Mechanisms of Inflammation

Apply knowledge of the biochemistry and cellular physiology to describe pathogenic mechanisms of acute and chronic inflammation and the resulting pathology at the cellular, tissue, and organism levels.  

*Objective FLAM1.1: Acute Inflammatory Response*. Describe the time course of the vascular and cellular events responsible for the acute inflammatory response to injury and discuss the receptors and ligands that are responsible for these events.  

*Objective FLAM1.2: Phagocytosis*. Describe the cellular process of phagocytosis and the molecular mechanisms of intracellular killing.  

*Objective FLAM1.3: Mediators of Inflammation*. Discuss the chemical mediators of inflammation, classifying the mediators with respect to origins, targets, and mechanisms of action.  

*Objective FLAM1.4: Systemic Effects of Inflammation*. Describe systemic consequences of changes secondary to inflammation, including metabolic consequences of changes in levels of serum proteins (acute phase reactants) and other inflammatory mediators.  

*Objective FLAM1.5: Outcomes of Inflammation*. Summarize the possible pathological outcomes of inflammation and discuss factors that determine what outcomes are seen under different circumstances.  

*Objective FLAM1.6: Morphologic Patterns of Inflammation*. Recognize and classify the major types of inflammatory lesions that can be present in histologic sections and identify the cellular and protein constituents in these lesions.  

*Objective FLAM1.7: Acute, Chronic, and Granulomatous Inflammation*. Compare and contrast acute, chronic, and granulomatous inflammation with respect to the major cell type(s) involved in the processes, the types of etiologic agents that produce each of these, and the mechanisms of tissue injury seen with these different types of inflammation.  

*Objective FLAM1.8: Extravascular Fluids Associated with Injury*. Classify, with appropriate terminology, the types of extravascular fluids associated with injury based on their cellular and protein content and provide examples of pathologic conditions in which these can be found.

### Topic: Immunological Mechanisms (IM)

This topic includes the understanding of normal and dysregulated innate and adaptive cellular immune responses resulting in inflammation, resolution, and disease.  

#### Learning Goal 1: Immune Dysfunction

Apply knowledge of basic mechanisms of immunology to explain how dysfunction can produce cellular injury, acute and chronic inflammation, autoimmunity, allergic reactions, and susceptibility to infection; how these changes affect organ function and the health of the organism; and how therapeutic intervention can mitigate these effects.  

*Objective IM1.1: Innate and Adaptive Immunity*. Compare and contrast innate and adaptive immunity with respect to the molecules and cells involved in the immune response, and the role of these systems in host defense.  

*Objective IM1.2: Cell Types*. Compare and contrast the roles played by T cells, B cells, natural killer (NK) cells, macrophages, plasma cells, and dendritic cells in the immune response.  

*Objective IM1.3: Cytokines*. Discuss, with examples, the production of different cytokines by different immune cells, the roles that cytokines play in effecting the immune response, and how knowledge of cytokine action can be exploited in the treatment of disease.  

*Objective IM1.4: Hypersensitivity*. Compare and contrast the mechanisms of the 4 hypersensitivity reactions with respect to the situations in which each is triggered, mechanisms of injury, resulting pathologic effects on tissue, and the ultimate clinical consequences.  

*Objective IM1.5: Complement*. Discuss how the complement cascade is activated, the role its activation plays in both inflammation and cellular cytotoxicity, and how abnormalities in complement function can produce disease.  

*Objective IM1.6: Immune Tolerance*. Define immunological tolerance and describe the role that failure of tolerance plays in the development of autoimmune diseases.  

*Objective IM1.7: Human Leukocyte Antigen (HLA)*. Discuss the structure and function of human histocompatibility antigens and describe the role of this system in both transplantation and susceptibility to certain diseases.  

*Objective IM1.8: Tissue Transplantation*. Discuss the complications of tissue transplantation, including the risk of infection and neoplasia, and the pathophysiology and clinicopathologic findings of hyperacute, acute, and chronic rejection.  

*Objective IM1.9: Primary Immunodeficiencies*. Compare and contrast the genetic basis and inheritance patterns of the well-defined primary immunodeficiency syndromes, discuss the pathogenesis and clinical manifestations of these disorders, and describe therapeutic interventions that can mitigate or correct them.  

*Objective IM1.10: Secondary Immune Deficiencies*. Describe the etiology, mechanisms of action, and possible clinical consequences of secondary immune deficiencies.  

*Objective IM1.11: Bone Marrow Transplantation*. Discuss common complications of bone marrow allograft transplantation, including the pathophysiologic and clinicopathologic features of acute and chronic graft-versus-host disease.

### Topic: Infectious Mechanisms (FECT)

This topic includes the mechanisms by which microorganisms, viruses, and parasites cause disease, including virulence factors produced by microorganisms and host response.

#### Learning Goal 1: Mechanisms of Infection

Apply knowledge of biochemical and cellular physiology to describe the pathogenic mechanisms and clinical manifestations of infectious diseases, including both pathogen and host factors and the resulting pathology at the cellular, tissue, and organism levels.  

*Objective FECT1.1: Host Barrier*. Explain the human host barrier to infection and describe how organisms spread within the body once the barrier is broken.  

*Objective FECT1.2: Categories of Infective Agents*. Describe the general categories of infective agents, including bacteria, viruses, fungi, and parasites, and describe the morphologic patterns of infectious diseases and the general mechanisms by which each category causes disease.  

*Objective FECT1.3: Host Responses to Infection*. Compare and contrast host responses to different classes of infectious agents in terms of morphological features, mechanisms of action, and mechanisms of immune evasion.  

#### Learning Goal 2: Pathogenic Mechanism of Infection

Apply knowledge of biochemical and cellular physiology to describe pathogenic mechanisms; the resulting pathology at the cellular, tissue, and organism levels; and the clinical manifestations of viral, bacterial, fungal, and parasitic infections.  

*Objective FECT2.1: Viral Mechanisms*. Compare and contrast the mechanisms by which RNA, DNA, and retroviral viruses enter and damage cells.  

*Objective FECT2.2*: *Patterns of Viral Infection*. Compare and contrast viruses that result in acute transient, chronic latent, chronic productive, and transformative infections and discuss how these differences result in divergent disease pathogenesis.  

*Objective FECT2.3: Histopathologic Features of Viral Infection*. Compare and contrast the histopathological features of herpes virus, cytomegalovirus, human papilloma virus, and adenovirus and recognize these histopathological features in images of different tissues.  

*Objective FECT2.4: Mechanisms of Bacterial Damage*. Describe the mechanisms by which bacteria damage cells and tissues, comparing and contrasting mechanism characteristics of infection with categories of bacteria.  

*Objective FECT2.5: Transmission Patterns of Bacterial Infection*. Discuss the different patterns of transmission of bacterial diseases as a function of both the type of organism and the organ systems involved in the infection.  

*Objective FECT2.6: Tissue Response to Bacterial Infection*. Describe the histologic patterns of tissue response to bacterial infection as a function of differences in the organisms involved, the specific organ affected, and the manner by which the bacterium enters the organ.  

*Objective FECT2.7: Special Stains for Bacteria*. Moved to C3 SP1.4  

*Objective FECT2.8: Fungal Infection*. List the different types of fungal organisms that infect humans and compare and contrast the mechanisms by which they damage tissues, the inflammatory responses they induce, and the resultant diseases that arise.  

*Objective FECT2.9: Histopathologic Features of Fungal Infection*. Moved to C3 SP1.5  

*Objective FECT2.10: Fungal Infection in Immunosuppression*. Compare and contrast the types of fungal infections that occur in immunosuppressed and immunocompetent patients with respect to the organisms involved, the mechanisms of organ damage, and the resultant clinical manifestations.  

*Objective FECT2.11: Parasitic Infections*. Describe classes of parasites that produce human disease, give examples of each class, and discuss their life cycle within humans and within other hosts.  

*Objective FECT2.12: Tissue Response to Parasitic Infection*. Discuss the mechanisms of pathologic damage caused by different parasites in different tissues and describe the diseases, complications, and possible outcomes associated with such infections.  

*Objective FECT2.13: Histologic Features of Parasitic Infection*. Moved to C3 SP1.5

### Topic: **Tissue Renewal, Regeneration, and Repair (RRR)**

This topic includes an understanding of the pathogenesis of cellular proliferation for regeneration of cells, including of stem cells, and tissue as well as knowledge of signal mechanisms for the repair process.

#### Learning Goal 1: Mechanisms of Tissue Regeneration, Renewal, and Repair

Apply knowledge of biochemistry and cellular physiology to describe the pathogenic mechanisms of tissue regeneration, renewal, and repair; the resulting pathology at the cellular, tissue, and organism levels; and clinical manifestations.  

*Objective RRR1.1: Stem Cells*. Compare and contrast embryonic and adult (somatic) stem cells with respect to their ability to proliferate and differentiate into different cell types; define induced pluripotent stem cells and compare and contrast them with the other types of stem cells.  

*Objective RRR1.2: Cell Cycles*. Describe the 5 stages of the cell cycle and explain the role of cyclins, cyclin-dependent kinase, and other proteins in the regulation of progression through the cell cycle and how disruption of the cell cycle can lead to disease.  

*Objective RRR1.3: Signaling Pathways*. Discuss the major signaling pathways involved in the regulation of cell growth, listing important cell surface receptors and describing the mechanisms whereby engagement of receptors by growth factors leads to cell growth.  

*Objective RRR1.4: Extracellular Matrix*. List the important proteins of the extracellular matrix, describe the role of cell–matrix interactions in cell growth and differentiation, and provide examples of how structural alterations of matrix proteins produce disease.  

*Objective RRR1.5: Angiogenesis*. Describe the regulation of angiogenesis, discussing receptors on vascular endothelium as well as the role soluble and stromal factors play in the process, and describe the effect of aberrant angiogenesis in disease.  

*Objective RRR1.6: Wound Healing*. Describe the phases of cutaneous wound healing, the mechanisms of healing by first intention (primary union) and second intention (secondary union), and possible clinical consequences of abnormal wound healing.  

*Objective RRR1.7: Anti-inflammatory Drugs and Wound Repair*. Explain the effects of anti-inflammatory medications on wound repair.

### Topic: **Hemodynamic Disorders and Thromboembolic Disease (HDTD)**

This topic includes a basic knowledge of edema, congestion, and shock as well as a basic understanding of the coagulation cascade to understand the pathogenesis of thromboembolic disorders.  

#### Learning Goal 1: Hemodynamics and Shock

Apply knowledge of biochemical and cellular physiology to discuss the pathogenic mechanisms resulting in alterations in hemodynamics and shock. Describe the resulting pathology at the cellular, tissue, and organism level and describe clinical manifestations associated with these pathologic changes.  

*Objective HDTD1.1: Edema*. Describe the pathophysiologic categories of edema and compare and contrast, with examples, how edema can be produced as a result of changes in hydrostatic pressure or plasma oncotic pressure.  

*Objective HDTD1.2: Hyperemia and Hemorrhage*. Explain the clinical, morphological, and physiological significance of hyperemia, congestion, and hemorrhage with respect to the disease states that cause them.  

*Objective HDTD1.3: Shock*. Classify different types of shock according to etiology and compare and contrast the pathogenesis of these different types.  

#### Learning Goal 2: Clotting and Disruption of Blood Flow

Apply knowledge of the biochemical and cellular physiology to discuss pathogenetic mechanisms that result in alterations in blood clotting or other disruptions to blood flow. Describe the resulting pathology at the cellular, tissue, and organism level and the clinical manifestations associated with these pathologic changes.  

*Objective HDTD2.1: Blood Clotting*. Discuss the vascular, cellular, and humoral events involved in blood clotting, including factors that involve primary and secondary hemostasis and antithrombotic regulation, and provide examples of genetic or acquired factors that can lead to either excess clotting or bleeding.  

*Objective HDTD2.2: Thrombosis and Thromboembolism*. Compare and contrast mechanism of thrombosis in situ and thromboembolism with respect to sites of involvement, risk factors, and attendant pathologic and clinical consequences.  

*Objective HDTD2.3: Embolism*. Describe the mechanism of embolus formation from different sources and compare and contrast the clinical settings and consequences of each.  

*Objective HDTD2.4 (Formerly C2HPCD2.5): Mechanisms of Hypercoagulability*. Compare and contrast the roles of endothelial injury, stasis, and alterations in the regulation of blood clotting in the development of the hypercoagulable state.  

*Objective HDTD2.5 (Formerly C2 HPCD1.1): Platelets in Hemostasis*. Summarize the role played by platelets in hemostasis, including platelet adhesion, activation, and aggregation.

### Topic: **Adaptation and Cell Death (ACD)**

This topic includes a basic understanding of the cellular responses to stress, mechanisms of cellular injury, and differentiation of necrosis and apoptosis.  

#### Learning Goal 1: Cellular Response to Injury

Apply knowledge of membrane physiology, metabolism, signal transduction, and macromolecular synthesis to discuss cellular responses to injury at the cell, tissue, and organism levels; how these responses affect morphologic appearance; and how they can be used for diagnostic, prognostic, and therapeutic purposes.  

*Objective ACD1.1: Adaptation*. Discuss the pathogenesis of hyperplasia, hypertrophy, atrophy, and metaplasia and compare and contrast their possible physiologic and pathologic causes.  

*Objective ACD1.2*: *Necrosis*. Define necrosis and compare and contrast the forms of necrosis produced in response to different etiologic agents with respect to their variable clinical and morphologic features.  

*Objective ACD1.3*: *Ischemia*. Compare and contrast ischemia and hypoxia and discuss the time course of the molecular events that occur in a cell in response to lack of oxygen, emphasizing the events that distinguish reversible from irreversible injury.  

*Objective ACD1.4: Reperfusion Injury*. Summarize the cell's response to reperfusion injury, emphasizing how reperfusion can exacerbate injury produced by ischemia.  

#### Learning Goal 2: Cell Death

Apply knowledge of biochemistry and cellular physiology to differentiate between pathogenic and physiologic mechanisms of cell death, the resulting morphologic appearance, and the physiologic and clinical settings in which these mechanisms are activated.  

*Objective ACD2.1: Apoptosis*. Contrast the etiology, mechanisms, and morphologic changes of apoptosis with those of necrosis. Discuss the circumstances in which dysregulation of apoptosis can produce disease and circumstances that determine why cells undergo apoptosis vs. necrosis.  

#### Learning Goal 3: Sublethal Injury

Apply knowledge of cellular physiology, metabolism, and macromolecular synthesis to discuss cellular and subcellular responses to sublethal injury or stress on cells; how these responses affect morphologic appearance at the cell and tissue level; and how they can affect organ function.  

*Objective ACD3.1: Cellular Response to Stress or Injury*. Discuss, with examples, the pathologic changes that occur in cellular organelles or cytoskeletal proteins of different cell types in response to stress or injury.  

*Objective ACD3.2: Intracellular Accumulations*. Describe the mechanisms of intracellular accumulations and the morphologic and clinical consequences of these accumulations.

### Topic: **Developmental Processes (DEV)**

This topic includes a basic knowledge of common morphologic abnormalities to understand the pathogenesis of developmental disorders.

#### Learning Goal 1: Development

Apply knowledge of embryology to describe the anatomy and physiology of developmental defects and problems related to prematurity.  

*Objective DEV1.1: Developmental Defects*. Describe, with examples, the pathogenesis and pathologic features of disorders related to defects in development.  

*Objective DEV1.2: Gestational Prematurity*. Discuss, with examples, the pathogenesis and pathologic features of disorders related to gestational prematurity.

### Topic: **Geriatrics (GER)**

This topic includes a basic knowledge of cellular physiology to understand the pathogenesis of aging.  

#### Learning Goal 1: Mechanisms of Aging

Apply knowledge of cellular physiology and biochemistry to describe the pathogenic mechanisms of aging.  

*Objective GER1.1: Aging*. Describe the pathogenesis of aging at the cellular level, discussing decreased cellular function, cellular damage and accumulations, and decreased DNA repair.  

## Competency 2

### Organ System Pathology

Once the student has mastered the fundamental mechanisms and processes for causing, sustaining, extending, or resolving injury, this knowledge can be integrated to understand how pathology in each organ system affects the initial pathologic site, multi-organ systems, and the overall function of the patient.

There are 23 topics within this competency area. Each topic includes general learning goals and specific objectives that medical students should be able to meet upon graduation from medical school. Table 2 lists the topic areas and shows the number of goals and objectives for each.

**Table 2:** Organ system pathology.

Topic	Number of Goals	Number of Objectives	Reference Code
Cardiovascular: Blood vessels	4	13	CBV
Cardiovascular: Heart	8	24	CH
Hematopathology: Red cell disorders	2	8	HRC
Hematopathology: White cell disorders	7	29	HWC
Hematopathology: Platelets and coagulation disorders	2	16	HPCD
Respiratory system	7	38	RS
Head and neck	3	21	HN
Gastrointestinal tract	7	33	GT
Hepatobiliary	9	34	HB
Pancreas	2	6	P
Kidney	5	23	UTK
Bladder	3	11	UTB
Male reproductive: penile	2	4	MRP
Male reproductive: Prostate	2	6	MP
Male reproductive: Testes	2	5	MT
Breast	2	12	BR
Female reproductive: Uterus, cervix, and vagina	4	14	FU
Female reproductive: Ovary	2	6	FO
Female reproductive: Disorders of pregnancy	1	7	FDP
Endocrine	7	22	EN
Skin	6	17	SK
Musculoskeletal system	3	17	MS
Nervous system: Central nervous system	9	37	NSC
Nervous system: Peripheral nervous system and eye	3	10	NSP
Autoimmune and multisystem disorders	1	4	AIMS

### Topic: **Cardiovascular—Blood Vessels (CBV)**

Cardiovascular disorders resulting from abnormal development, hypoxia, immune dysregulation, infections, neoplasms, and smooth muscle changes as they relate to the blood vessels are enumerated.  

#### Learning Goal 1: Mechanisms of Atherosclerosis

Apply knowledge of immunologic principles, inflammation, and tissue repair to explain atherosclerosis and its complications.  

*Objective CBV1.1: Factors Contributing to Endothelial Injury*. Explain how environmental factors (including elevated cholesterol and low-density lipoprotein complexes, infection, and smoking) can contribute to endothelial cell injury.  

*Objective CBV1.2: Feedback in Endothelial Damage*. Describe the positive feedback loop in which damaged endothelial cells cause further endothelial damage.  

*Objective CBV1.3: Atherosclerotic Plaque Rupture*. Predict the local and distant consequences that are likely to follow rupture of an atherosclerotic plaque and the resultant clinical presentation.  

*Objective CBV1.4: Vascular Aneurysm*. Describe the morphologic changes in atherosclerosis and discuss how atrophic changes in the vessel wall may result in aneurysm formation.  

*Objective CBV1.5: Pathogenesis of Atherosclerosis*. Describe the pathogenesis of atherosclerotic plaque formation and the different lesions that may be seen at various stages as well as most common vessels in which it develops.  

#### Learning Goal 2: Vascular Aneurysms and Dissection

Apply knowledge of the structure and components of blood vessels, vascular physiology, and basic hemodynamic principles to explain the development, manifestations, and consequences of vascular aneurysms and dissection.  

*Objective CBV2.1: Thrombus Formation*. Deleted. Use C1 HDTD2.2.  

*Objective CBV2.2: Aortic Aneurysm and Dissection*. Compare and contrast aortic aneurysms and aortic dissections in terms of their predisposing factors, the sites of involvement, morphology, and clinical consequences.  

*Objective CBV2.3: Abdominal Aortic Aneurysm*. Retired. Use C2 CBV2.2.  

#### Learning Goal 3: Vasculitis

Apply knowledge of microbiological principles and mechanisms of immunologically mediated disease to discuss the pathogenesis, clinical presentation, morphological features, and laboratory diagnosis of the different vasculitides.  

*Objective CBV3.1: Drug-induced Vasculitis*. Describe the pathogenesis of a drug-induced vasculitis.  

*Objective CBV3.2: Autoimmune Vasculitis*. Compare and contrast the mechanisms by which an autoimmune disease can appear as a vasculitis in one specific organ or as a generalized disease in many organs.  

*Objective CBV3.3: Vasculitis of Large and Medium-sized Vessels*. Compare and contrast the vasculitides that occur in large- and medium-sized vessels.  

*Objective CBV3.4: Vasculitis of Small Vessels*. Compare and contrast the vasculitides that occur in small vessels.  

*Objective CBV3.5: Infectious Vasculitis*. Discuss the pathogenesis of common bacterial or fungal agents that cause vasculitis.  

#### Learning Goal 4: Vascular Neoplasms and Malformations

Apply knowledge of vascular structure and function and hemodynamics to discuss the pathogenesis, clinical presentation, morphological features, and diagnosis of the different vascular neoplasms.  

*Objective CBV4.1: Benign or Reactive Vascular Disorders*. Compare and contrast benign vascular neoplasms, vascular malformations, and vascular ectasia.  

*Objective CBV4.2: Vascular Neoplasms*. Compare and contrast intermediate-grade and malignant vascular neoplasms such as Kaposi sarcoma and angiosarcoma.

### Topic: Cardiovascular—Heart **(CH)**

Cardiovascular disorders resulting from abnormal development, hypoxia, immune dysregulation, infections, neoplasms, and intrinsic muscle disease as they relate to the heart are enumerated.  

#### Learning Goal 1: Heart Failure

Apply knowledge of anatomy, physiology, and general pathophysiologic principles to describe the pathogenesis and clinical and morphologic features of heart failure.  

*Objective CH1.1: Right- and Left-Sided Heart Failure*. Compare and contrast right heart failure versus left heart failure in terms of pathogenesis, clinical features, pathologic features, and the short-term and long-term consequences.  

*Objective CH1.2: Cardiomyopathy*. Moved to C2 CH1.2.  

#### Learning Goal 2: Atherosclerosis in Heart Disease

Apply knowledge of anatomy, physiology, and general pathophysiologic principles to explain how atherosclerosis leads to heart disease and death.  

*Objective CH2.1: Ischemic Heart Disease*. Explain how ischemic heart disease can progress while remaining entirely free of symptoms for many years.  

*Objective CH2.2: Angina*. Contrast the clinical, physiologic, and morphologic differences between stable angina and unstable angina.  

*Objective CH2.3: Reperfusion Versus Ischemic Injury*. Contrast the behavior of the myocardium that has been subjected to chronic ischemia alone from that of reperfused myocardium following therapy for infarction.  

*Objective CH2.4: Timing of Changes in Myocardial Infarction*. Compare and contrast the gross and microscopic features and pathophysiologic changes of acute myocardial infarction through remote myocardial infarction, describing the spectrum of changes that occur at specific times.  

*Objective CH2.5: Histopathology of Myocardial Infarction*. Retired, now combined into C2 CH2.4  

*Objective CH2.6: Complications of Myocardial Infarction*. Identify short-term and long-term complications of myocardial infarction.  

#### Learning Goal 3: Cardiovascular Malformation

Apply knowledge of embryologic principles to describe how improper development of the heart and blood vessels leads to cardiac dysfunction.  

*Objective CH3.1: Congenital Heart Disease*. Name the most common forms of congenital heart disease and outline their clinical presentation, natural history, and long- and short-term complications.  

*Objective CH3.2: Congenital Heart Disease Associated with Genetic Disorders*. Name several common genetic disorders associated with congenital heart disease, describing the clinical presentations and pathogenesis.  

*Objective CH3.3: Paradoxical Embolism*. Describe a paradoxical embolus and its relationship to congenital heart disease.  

*Objective CH3.4: Cardiac Shunts*. Define the concepts of left-to-right shunt, right-to-left shunt, and shunt reversal and correlate with clinical presentation.  

#### Learning Goal 4: Cardiac Inflammatory Conditions

Apply knowledge of immunological and microbiological principles to explain the role of infectious agents and inflammatory conditions in myocardial dysfunction and describe the related clinical presentations.  

*Objective CH4.1: Rheumatic Fever*. Describe the major cardiac and extracardiac manifestations of rheumatic fever, including its effect on the endocardium, myocardium, and pericardium.  

*Objective CH4.2: Rheumatic Fever and Endocarditis*. Compare and contrast the effects of rheumatic fever and bacterial endocarditis on the heart.  

*Objective CH4.3: Infective Endocarditis*. Describe the two major patterns of infective endocarditis and the pathologic changes seen in the cardiac valves.  

*Objective CH4.4: Noninfective Endocarditis*. Discuss the pathologic features of noninfective endocarditis on the cardiac valves.  

*Objective CH4.5: Myocarditis*. Describe the clinicopathologic features and common causes of myocarditis and their consequences.  

*Objective CH4.6: Pericarditis*. Summarize the common causes of pericarditis and the resultant clinicopathologic features.  

#### Learning Goal 5: Valvular Dysfunction

Apply knowledge of the anatomy and physiology of heart valves to explain how valvular dysfunction leads to heart failure and describe the related clinical presentation.  

*Objective CH5.1: Valve Stenosis*. Discuss the underlying causes, clinicopathologic features, and complications associated with cardiac valvular stenosis.  

*Objective CH5.2: Valve Insufficiency*. Describe the etiologies, clinicopathologic features, and complications of cardiac valvular insufficiency.  

*Objective CH 5.3: Valve Dysfunction*. Compare and contrast valvular stenosis and insufficiency. Outline cardiac disorders that lead to either.  

#### Learning Goal 6: Hypertension

Apply knowledge of the mechanism of hypertension and how tissues respond to increased resistance to describe the clinical and pathologic changes seen in systemic and pulmonary hypertension.  

*Objective CH6.1: Cardiac Changes in Pulmonary Hypertension*. Describe the pathogenesis and the gross and microscopic adaptive changes in the myocardium that result from pulmonary hypertension.  

*Objective CH6.2: Cardiac Changes in Systemic Hypertension*. Discuss the pathogenesis and the gross and microscopic adaptive changes in the myocardium that result from systemic hypertension.  

*Objective CH6.3: Mechanisms of Hypertension*. Describe the mechanisms and epidemiology of primary hypertension and discuss the causes of secondary hypertension.  

#### Learning Goal 7: Cardiomyopathy

Apply knowledge of the mechanism of myocardial function to describe the clinicopathologic features of cardiac and systemic disorders that lead to cardiac myocyte dysfunction.  

*Objective CH7.1 (Formerly C2 CH1.1): Cardiomyopathy*. Compare and contrast the clinicopathologic features of dilated, restrictive, and hypertrophic cardiomyopathies.  

#### Learning Goal 8: Cardiac Neoplasms

Apply knowledge of the molecular basis of neoplasia to describe the pathogenesis, clinical presentation, biologic behavior, morphologic appearance, classification, diagnosis, and prognosis of neoplasms affecting the heart.  

*Objective CH8.1: Cardiac Neoplasms*. Compare and contrast the pathogenesis and clinicopathologic features of primary and secondary cardiac tumors.

### Topic: Hematopathology—Red **Cell Disorders (HRC)**

Red blood cell (RBC) disorders resulting from abnormal development, nutritional derangements, inherited disorders, blood loss, and intrinsic disease as they relate to anemia and polycythemia.  

#### Learning Goal 1: Anemia

Apply knowledge of nutritional biochemistry, erythropoiesis, and RBC structure and function to a discussion of the behavioral, hereditary, developmental, and chronic causes of anemia.  

*Objective HRC1.1: Iron Deficiency and RBC Development*. Explain the contribution of iron to RBC development and function. Describe behaviors and conditions that lead to iron deficiency and contrast the morphology and laboratory parameters of normal red cells versus iron-deficient cells.  

*Objective HRC1.2: Hereditary Spherocytosis*. Discuss the pathophysiology, morphology, and clinical features of hereditary spherocytosis.  

*Objective HRC1.3: Hepcidin Regulation, Iron Overload, and Anemia of Chronic Disease*. Discuss the role of hepcidin as an iron regulator and describe how different types of alterations in the hepcidin pathway can produce anemia of chronic disease or iron overload.  

*Objective HRC1.4: B*_*12*_*and Folate Deficiencies*. Discuss the role of vitamin B_12_ and folic acid in red cell development and describe the pathophysiology of anemia arising from B_12_ and folic acid deficiency.  

*Objective HRC1.5: Anemias of Red Cell Destruction*. Explain the mechanisms by which anemia is produced on the basis of shortened red cell survival, distinguishing between intrinsic and extrinsic causes of red cell destruction.  

*Objective HRC1.6: Aplastic Anemia*. Retired. Use C2 HWC5.6  

*Objective HRC1.7: Hemoglobinopathies and Thalassemia*. Describe the structural alterations and regulatory abnormalities associated with hemoglobinopathies and thalassemia and discuss how these abnormalities give rise to the clinical manifestations of these diseases.  

*Objective HRC1.8: Blood Loss*. Discuss the pathogenesis and clinicopathologic features of anemia associated with acute and chronic blood loss.  

#### Learning Goal 2: Polycythemia

Apply knowledge of physiology, erythropoiesis, environmental factors, and RBC structure and function to a discussion of the causes of polycythemia.  

*Objective HRC2.1: Polycythemia*. Describe the pathogenesis and clinicopathologic features of disorders resulting in increased red cell mass (primary and secondary polycythemia) and contrast to relative polycythemia.

### Topic: Hematopathology—White **Cell Disorders, Lymph Nodes, Spleen, and Thymus (HWC)**

Disorders of white blood cells and hematolymphoid tissues resulting from abnormal development, genetic mutations, neoplasms, infections, and intrinsic disease as they relate to reactive and neoplastic abnormalities are enumerated.  

#### Learning Goal 1: Development of White Blood Cells and Non-neoplastic Causes of Neutropenia

Apply knowledge of anatomy, histology, and physiology to describe the normal development of white blood cells and non-neoplastic conditions leading to increased or decreased numbers of white blood cells.  

*Objective HWC1.1: Morphology of White Cells*. Moved to C2 HWC5.4.  

*Objective HWC1.2: White Cell Growth Factors*. Moved to C2 HWC5.5.  

*Objective HWC1.3: Leukocytosis*. Define leukocytosis and describe etiologies to include those causing neutrophilia, lymphocytosis, monocytosis, eosinophilia, basophilia, and a leukemoid reaction.  

*Objective HWC1.4: Leukopenia*. Compare and contrast the causes, mechanisms, and consequences of leukopenia including discussion of neutropenia and lymphopenia and compare with those of pancytopenia.  

*Objective HWC1.5: Neutrophilia*. Retired. Now combined in C2 HWC1.3.  

*Objective HWC1.6: Neutropenia*. Retired. Now combined in C2 HWC1.4.  

#### Learning Goal 2: Genetic Mutations in Hematologic Malignancy

Apply knowledge of general concepts of neoplasia to explain how genetic mutations can produce hematologic malignancies and how the clinical behavior of different malignancies can be explained by different mutations.  

*Objective HWC2.1: Germline and Somatic Mutations in Hematologic Malignancy*. Explain the difference between germline and somatic mutations; give examples and explain how each mutation contributes to the development of hematologic malignancies.  

*Objective HWC2.2: Translocations in Oncogenes*. Compare and contrast, with examples, translocations that result in hematologic malignancy by activation of oncogenes with those that produce fusion proteins.  

*Objective HWC2.3: Cell Proliferation or Cell Death in Lymphomas*. Explain, with examples, how dysregulation of cell proliferation or of cell death can give rise to lymphomas and compare and contrast the morphologic appearance and clinical behavior of diseases arising by each mechanism.  

*Objective HWC2.4: Molecular Basis of Leukemia and Lymphoma*. Describe how understanding the molecular pathogenesis of leukemia and lymphoma can suggest targets for therapeutic intervention and give examples of diseases currently treated by targeted therapy.  

*Objective HWC2.5: Multiple Myeloma*. Moved to C2 HWC3.7.  

*Objective HWC2.6: Clonal Hematopoiesis*. Define clonal hematopoiesis, discuss its relationship to bone marrow disorders, and describe some of its systemic consequences.  

#### Learning Goal 3: Classification of Leukemia and Lymphomas

Apply knowledge of hematopoiesis to discuss the pathophysiologic basis for the classification of leukemia and lymphomas.  

*Objective HWC3.1: Morphology of Acute Leukemia and Lymphoma*. Describe the morphologic features that characterize typical cases of acute leukemia and lymphoma.  

*Objective HWC3.2: Myeloid Neoplasia*. Compare and contrast myelodysplastic syndromes, myeloproliferative neoplasms, and acute myeloid leukemia with respect to morphologic appearance, clinical features, and underlying pathophysiology.  

*Objective HWC3.3: Categories of Lymphoma*. Compare and contrast low-grade or indolent lymphomas and high-grade or aggressive lymphomas with respect to morphologic appearance, clinical features, and underlying pathophysiology.  

*Objective HWC3.4: Morphology of Acute and Chronic Leukemia*. Compare and contrast the morphologic appearance of myeloblasts, lymphoblasts, and mature lymphocytes and distinguish acute myeloid leukemia from chronic myeloid leukemia.  

*Objective HWC3.5: Morphology of Lymphomas*. Describe the histologic appearance of typical cases of follicular lymphoma, diffuse large B-cell lymphoma, small lymphocytic lymphoma/chronic lymphocytic leukemia, and Hodgkin lymphoma.  

*Objective HWC3.6: Hodgkin and Non-Hodgkin Lymphoma*. Compare and contrast Hodgkin lymphoma with at least 2 non-Hodgkin lymphomas with respect to age and clinical symptoms at presentation, sites and pattern of spread of disease, cell of origin, histologic appearance, and prognosis and response to therapy.  

*Objective HWC3.7 (Formerly C2 HWC2.5): Multiple Myeloma*. Describe the clinicopathologic features of multiple myeloma in terms of clinical presentation, laboratory findings, radiologic findings, histologic features, and prognosis.  

#### Learning Goal 4: Clinical Features of Hematolymphoid Neoplasms

Discuss the clinical manifestations of hematolymphoid neoplasms, including age distribution of different tumors, presenting symptoms and signs, disease complications, natural history, and response to therapy.  

*Objective HWC4.1: Clinical Features of Bone Marrow Neoplasms*. Identify the tumors of bone marrow most likely to present with anemia, leukopenia, or thrombocytopenia and discuss the presenting clinical features most likely to be associated with each.  

*Objective HWC4.2: B Symptoms in Hematolymphoid Neoplasia*. Define B symptoms, list which lymphomas are most and least likely to be associated with them, and discuss the prognostic implications of B symptoms in these diseases.  

*Objective HWC4.3: Staging of Hematolymphoid Neoplasia*. Define staging as it applies to lymphoma and give examples of different lymphomas in which staging has different clinical implications.  

*Objective HWC4.4: Extranodal Lymphoma*. Identify lymphomas most likely to present in or involve extranodal sites such as the gastrointestinal tract (GT), bone marrow, blood, skin, or central nervous system.  

#### Learning Goal 5: Stem Cells and Hematologic Development and Hematolymphoid Neoplasia

Describe how stem cells give rise to the diverse cell populations seen in bone marrow and lymph nodes and discuss how knowledge of hematopoietic cell development can provide a framework for understanding hematolymphoid neoplasia.  

*Objective HWC5.1: Cell of Origin and the Morphology of Neoplasia*. Outline, with examples, the difference between the cell of origin of a neoplasm and the morphologic expression of that disease.  

*Objective HWC5.2: Stem Cells in Myeloid Leukemias*. Discuss the evidence that supports the existence of stem cells in myeloid leukemias and list the features of chronic myeloproliferative neoplasms that suggest they are derived from stem cells.  

*Objective HWC5.3: Lymphoid Response to B-Cell Activation*. Describe the morphologic and molecular changes that take place within a lymph node in response to B-cell activation and explain how these changes relate to different types of B-cell non-Hodgkin lymphoma.  

*Objective HWC5.4 (Formerly C2 HWC1.1): Morphology of White Cells*. Describe the maturational pathway of white blood cells, naming and describing the morphology of the cells present at each stage for each white blood cell type.  

*Objective HWC5.5(Formerly C2 HWC1.2): White Cell Growth Factors*. Define the role of growth factors in the development and maturation of white blood cells and how these are altered in reactive processes.  

*Objective HWC5.6: Bone Marrow Failure*. Compare and contrast the genetic, environmental, infectious, immunologic, and neoplastic causes of bone marrow failure.  

#### Learning Goal 6: Thymus

Apply knowledge of the anatomy and function of the thymus to summarize how developmental anomalies, immune disorders, and malignant transformation of epithelial and lymphoid cells lead to consequences such as immune dysfunction.  

*Objective HWC6.1: Thymic Neoplasms*. Describe the clinicopathologic features of thymic neoplasms and contrast these with lymphomas involving the mediastinum.  

*Objective HWC6.2: Thymic Development*. Explain how deficits in particular stages of thymic development can produce specific types of disease.  

*Objective HWC6.3: Paraneoplastic Conditions with Thymoma*. Discuss the clinical features of the paraneoplastic conditions associated with thymomas.  

#### Learning Goal 7: Spleen

Apply knowledge of the anatomy and function of the spleen to explain how developmental anomalies, immune and metabolic disorders, and neoplasia lead to splenic dysfunction.  

*Objective HWC7.1: Splenic Function.* Explain the contribution of normal splenic function to non-neoplastic diseases.  

*Objective HWC7.2: Splenomegaly*. Describe the clinical features, causes, and pathologic findings of neoplastic and non-neoplastic disorders leading to splenomegaly.

### Topic: Hematopathology—Platelets and **Coagulation Disorders (HPCD)**

Platelet disorders resulting from abnormal development, inherited disorders, acquired disorders, immune mechanisms, and infectious diseases and their central role in blood clotting as they relate to coagulation and hemostasis abnormalities are enumerated.  

#### Learning Goal 1: Platelets

Apply knowledge of platelet structure and function to discuss qualitative and quantitative disorders leading to abnormal bleeding.  

*Objective HPCD1.1: Platelets in Hemostasis*. Summarize the role played by platelets in hemostasis, including platelet adhesion, activation, and aggregation.  

*Objective HPCD1.2: Thrombocytopenia*. Identify the examples of each of the following pathogenetic categories of thrombocytopenia: decreased production, decreased platelet survival, sequestration, and dilutional effect.  

*Objective HPCD1.3: Thrombocytopenic Syndromes*. Compare and contrast the following thrombocytopenia syndromes: immune thrombocytopenic purpura (ITP), drug-induced thrombocytopenia, and heparin-induced thrombocytopenia.  

*Objective HPCD1.4: Thrombotic Thrombocytopenic Purpura (TTP)*. Compare and contrast TTP with hemolytic uremic syndrome.  

*Objective HPCD1.5: Platelet Disorders*. Explain the biochemical basis of the following congenital and acquired defective platelet disorders: Bernard-Soulier syndrome, Glanzmann thrombasthenia, storage pool disorders, aspirin-related dysfunction, and uremia-related dysfunction.  

*Objective HPCD1.6: Bone Marrow Aplasia*. Explain the bases of marrow aplasia/myelophthisis, nutritional deficiency, and myelodysplasia as causes of thrombocytopenia from marrow failure.  

#### Learning Goal 2: Hemostasis

Apply knowledge of normal hemostasis, interaction of platelets, and procoagulant and anticoagulant factors to describe qualitative and quantitative disorders leading to abnormal bleeding and thrombosis.  

*Objective HPCD2.1: Types of Hemorrhage*. Distinguish among the following manifestations of hemorrhage: hematoma, petechiae, purpura, and ecchymoses.  

*Objective HPCD2.2: Stages of Hemostasis*. Retired. Use C1 HDTD2.1.  

*Objective HPCD2.3: Secondary Hemostasis*. Retired. Use C1 HDTD2.1.  

*Objective HPCD2.4: Proteases and the Coagulation Cascade*. Retired. Use C1 HDTD2.1.  

*Objective HPCD2.5: Mechanisms of Hypercoagulability*. Retired. Use C1 HDTD2.4.  

*Objective HPCD2.6: Risk Factors for Thrombophilia*. Give examples and discuss the pathophysiology of inherited versus acquired conditions that increase the risk of thrombophilia.  

*Objective HPCD2.7: Disseminated Intravascular Coagulation*. Discuss disseminated intravascular coagulation in terms of etiologies, pathogenesis, clinical presentation, and course.  

*Objective HPCD2.8: Inherited Hemophilia*. Discuss the pathogenesis and clinical manifestations of hemophilia A and explain how it differs from hemophilia B.  

*Objective HPCD2.9: Vitamin K and Liver Disease*. Describe the pathogenesis and clinical findings in coagulopathy due to liver disease and vitamin K deficiency.  

*Objective HPCD2.10: von Willebrand Disease*. Compare and contrast types I, II, and III von Willebrand disease and explain the quantitative or qualitative abnormalities and features observed in each type.  

*Objective HPCD2.11: Antiphospholipid Antibody Syndrome*. Describe the pathogenesis and clinical findings in antiphospholipid antibody syndrome.  

*Objective HPCD2.12: Heparin-induced Thrombocytopenia*. Explain the mechanism of heparin-induced thrombocytopenia/thrombosis and describe its clinical presentation and approach to therapy.  

*Objective HPCD2.13: Thrombophilia in Cancer*. Explain the risk of thrombophilia in cancer, describe the context of Trousseau syndrome, and give examples of malignancies frequently associated with thrombophilia.

### Topic: Respiratory System **(RS)**

Respiratory disorders resulting from abnormal development, genetic mutations, immune mechanisms, infections, neoplasms, and intrinsic disease as they relate to lung abnormalities are enumerated.  

#### Learning Goal 1: Vascular Diseases of the Lung

Apply knowledge of the structure and function of blood vessels and pulmonary vascular physiology to explain the pathogenesis, clinical manifestations, and pathologic findings in disorders affecting the pulmonary vasculature.  

*Objective RS1.1: Pulmonary Embolism*. Compare and contrast the clinical manifestations, radiographic and pathologic findings, and potential consequences of pulmonary embolism in terms of single versus multiple and small versus large emboli.  

*Objective RS1.2: Conditions Predisposing to Pulmonary Embolism*. Discuss the factors, including underlying conditions, which can impact the incidence and clinical significance of pulmonary embolism.  

*Objective RS1.3: Pulmonary Hypertension.* Describe the structural cardiopulmonary conditions that are frequently associated with pulmonary hypertension.  

*Objective RS1.4: Conditions Contributing to Pulmonary Hypertension.* Explain how each of the following cardiopulmonary conditions contributes to pulmonary hypertension: increased pulmonary blood flow or pressure, increased pulmonary vascular resistance, and left heart resistance to blood flow.  

*Objective RS1.5: Pathogenesis of Pulmonary Hypertension.* Describe the pathogenesis of pulmonary hypertension in hereditary and secondary forms and the characteristic gross and microscopic morphologic features of each.  

*Objective RS1.6: Goodpasture Syndrome and Granulomatosis with Polyangiitis*. Compare and contrast the clinical manifestations, pathogenesis, and pathologic findings of Goodpasture syndrome and granulomatosis with polyangiitis (formerly known as Wegener granulomatosis).  

*Objective RS1.7: Pulmonary Edema*. Outline the pathogenesis and clinicopathologic features of disorders presenting with pulmonary edema and common conditions in which it occurs.  

*Objective RS1.8: Acute Lung Injury*. Discuss the pathogenesis and clinicopathologic features of acute lung injury and common settings in which it develops.  

#### Learning Goal 2: Pulmonary Infection

Apply knowledge of the local pulmonary defense mechanisms and systemic host resistance to infection to discuss pathogenesis, classification, clinical manifestations, and pathologic findings in lower respiratory tract infections in immunocompetent and immunocompromised hosts.  

*Objective RS2.1: Pulmonary Infections in the Immunocompromised Patient*. Discuss the common infectious agents that produce pulmonary disease that are generally associated with defects in innate, humoral, or cell-mediated immunity.  

*Objective RS2.2: Classification of Pneumonia by Setting*. Describe the classification of pneumonias by clinical setting and name the common etiologic agents for each category.  

*Objective RS2.3: Clinicopathologic Features of Pneumonia*. Compare and contrast the clinical presentation and manifestations, gross and microscopic pathology, prognosis, and potential complications for each category of pneumonia.  

*Objective RS2.4: Patterns of Pneumonia*. Define bronchopneumonia, lobar pneumonia, and atypical pneumonia/interstitial pneumonitis and compare and contrast the common etiologic agents and pathologic findings for each.  

*Objective RS2.5: Tuberculosis*. Compare and contrast the clinical presentation and gross and microscopic findings in primary, secondary/reactivation, and miliary tuberculosis.  

*Objective RS2.6: Influenza*. Retired. Use C3 MB3.2  

*Objective RS2.7: Upper and Lower Respiratory Viral Infections*. Compare and contrast the clinicopathologic findings in upper and lower respiratory tract viral infections.  

*Objective RS2.8: Aspiration Pneumonia*. Name risk factors for aspiration pneumonia and describe the pathology, prognosis, and potential complications.  

*Objective RS2.9: Lung Abscess*. Describe lung abscess in terms of pathogenesis, typical microorganisms, clinical presentation and course, and pathologic findings.  

*Objective RS2.10: Fungal Pneumonia*. Compare and contrast the causative agents, geographic locations, clinical presentation, and pathologic findings in chronic pneumonia caused by fungal organisms.  

*Objective RS2.11: Features of Pulmonary Infections in the Immunocompromised and Immunocompetent Host*. Discuss the differences in clinical presentation and the etiologic agents of pneumonia in immunocompetent versus immunocompromised hosts.  

*Objective RS2.12: Features of Upper and LowerRespiratory Infections*. Retired. Use C2 RS2.7.  

#### Learning Goal 3: Lung Neoplasia

Apply knowledge of neoplasia to describe the clinical presentation, pathophysiology, biologic behavior, morphologic appearance, classification, diagnosis, prognosis, and targeted therapy of lung neoplasms.  

*Objective RS3.1: Lung Neoplasms*. Describe the common locations for the different types of lung cancer.  

*Objective RS3.2: Morphologic Features of Lung Neoplasms*. Discuss key gross and histopathologic features that may help differentiate between small cell carcinoma, adenocarcinoma, and squamous cell carcinoma.  

*Objective RS3.3: Metastatic Carcinoma to the Lung*. Describe features that favor the diagnosis of metastatic carcinoma over a primary lung tumor.  

*Objective RS3.4: Genetics of Lung Cancer*. Describe the contribution of specific genetic mutations that are found in particular lung cancers and explain how these mutations affect therapeutic decisions.  

*Objective RS3.5: Environmental Factors Predisposing to Lung Cancer*. Explain the environmental factors that predispose to the development of lung cancer and illustrate how these factors interact with genetic factors in the development of cancer.

#### Learning Goal 4: Obstructive Diseases of the Lung

Apply knowledge of the genetic, immune, and environmental factors leading to cell injury to explain the clinical and pathophysiological consequences that result in obstruction to airflow.  

*Objective RS4.1: Emphysema*. Describe the role of smoking in emphysema; name the 4 different types of emphysema, which is most common, and which lobes of the lungs are most involved in centrilobular emphysema.  

*Objective RS4.2: Bronchiectasis*. Explain the gross morphologic changes associated with bronchiectasis and name at least 2 conditions that may lead to bronchiectasis.  

*Objective RS4.3:Pneumoconiosis*. Moved to C2 RS5.4.  

*Objective RS4.4: Asthma*. Compare and contrast the clinicopathologic features and causes of asthma and describe the morphologic changes and consequences that result in airflow obstruction.  

*Objective RS4.5: Bronchitis*. Compare and contrast the pathogenesis and clinicopathologic features of chronic bronchitis and describe the morphologic changes and consequences that result in airflow obstruction.  

*Objective RS4.6: Atelectasis*. Compare and contrast the pathogenesis and clinicopathologic features of the various disorders leading to atelectasis.

#### Learning Goal 5: Restrictive Diseases of the Lung

Apply knowledge of the factors leading to cell injury to explain the clinical and pathophysiological consequences that result in restrictive lung diseases.  

*Objective RS5.1: Pulmonary Fibrosis*. Describe the clinicopathologic features of lung disorders causing interstitial fibrosis.  

*Objective RS5.2: Granulomatous Disorders of the Lung*. Explain the pathogenesis and clinicopathologic features of non-infectious disorders manifested by granulomatous inflammation in the lungs.  

*Objective RS5.3: Pneumoconioses*. Explain the pathogenesis and clinicopathologic features of various common pneumoconioses, including the inciting substance and occupational or other route of exposure.  

*Objective RS5.4: Environmental Pollutants*. Compare and contrast the pathogenesis and clinicopathologic features of lung disorders due to air pollutants.  

*Objective RS5.5: Pulmonary Manifestations of Autoimmune Disorders*. Discuss the pathogenesis and clinicopathologic features of autoimmune disorders affecting the lung.  

*Objective RS5.6: Extrapulmonary Restrictive Disorders*. Outline the pathogenesis and clinicopathologic features of restrictive lung disorders due to causes outside the lung parenchyma (e.g., musculoskeletal disease, neuromuscular disease, obesity).

#### Learning Goal 6: Respiratory Disorders of the Fetus and Infant

Apply knowledge of the embryology of the respiratory tract and pulmonary development to outline the pathogenesis, morphological features, and clinical presentation of developmental anomalies and acquired newborn disorders.  

*Objective RS6.1: Respiratory Disorders of the Fetus and Infant*. Explain the pathogenesis and clinicopathologic features of congenital and acquired disorders affecting the airways and lungs of the fetus and infant.

#### Learning Goal 7: Pleural Disorders

Apply knowledge of the structure and function of pleura to explain the pathogenesis, clinical manifestations, and pathologic findings of pleural disorders.  

*Objective RS7.1: Pleural Cavity Non-neoplastic Lesions*. Outline the pathogenesis and clinicopathologic features of disorders affecting the pleural cavity (effusions, pneumothorax).  

*Objective RS7.2: Pleural Neoplasms*. Describe the clinicopathologic findings of neoplasms involving the pleura and pleural cavity.

### Topic: **Head and Neck (HN)**

Head and neck disorders resulting from abnormal development, genetic mutations, immune dysfunction, neoplasms, and intrinsic disease as they relate to the oral cavity, teeth, pharynx, nose, nasal cavity, sinuses, larynx, lower airways, ear, neck, and salivary glands are enumerated.  

#### Learning Goal 1: Non-neoplastic Salivary Gland Disorders

Apply knowledge of the structure and function of the salivary glands to an understanding of the pathogenesis and clinicopathologic features associated with disorders presenting with gland enlargement and/or dysfunction.  

*Objective HN1.1: Salivary Duct Obstruction*. Describe the potential causes for obstruction of the salivary gland duct and explain how a mucocele is formed.  

*Objective HN1.2: Sialadenitis*. Discuss inflammation of the salivary glands arising from trauma, infection, or autoimmune dysregulation and discuss their potential neoplastic complications.  

*Objective HN1.3: Sjogren Syndrome*. Describe Sjogren syndrome and discuss how it relates to salivary gland dysfunction, its effect on multiple organ systems, complications, and long-term risks.

#### Learning Goal 2: Head and Neck Neoplasia

Apply knowledge of the etiology, pathogenesis, morphological appearance, and classification of neoplasms involving the salivary glands, oral cavity, upper airways, neck, nose, sinuses, ear, and larynx to their diagnosis, prediction of biological behavior, prevention, and treatment.  

*Objective HN2.1: Benign and Malignant Tumors of Salivary Glands*. Compare and contrast the clinicopathologic and morphologic features of common benign and malignant tumors.  

*Objective HN2.2: Squamous Cell Carcinoma of the Oropharynx*. Discuss the etiology and pathogenesis of squamous cell carcinoma of the oropharynx and the spectrum of histologic findings from normal mucosa to invasive disease.  

*Objective HN2.3: Causes of Oropharyngeal Squamous Cell Carcinoma*. Retired, use C2 HN2.2.  

*Objective HN2.4: Developmental Neck Masses and Other Neck Tumors*. Revised and moved to C2 HN3.8.  

*Objective HN2.5: Odontogenic Tumors*. Discuss the pathogenesis and clinicopathologic features of odontogenic tumors.  

*Objective HN2.6: Neoplasms of the Nose, Nasopharynx and Sinuses*. Discuss the pathogenesis and clinicopathologic features of benign and malignant neoplasms involving the nose, nasopharynx, and sinuses (e.g., angiofibroma, sinonasal papilloma, olfactory neuroblastoma, midline carcinoma, nasopharyngeal carcinoma, and extranodal NK/T cell lymphoma).  

*Objective HN2.7: Laryngeal Neoplasia*. Describe the pathogenesis and clinicopathologic features of benign and malignant neoplasms involving the larynx and their precursor lesions.  

*Objective HN2.8: Ear Neoplasms*. Compare and contrast the pathogenesis and clinicopathologic features of benign and malignant neoplasms involving the ear.

#### Learning Goal 3: Non-neoplastic and Developmental Disorders Affecting the Oral Cavity, Nose, Nasal Cavity, Sinuses, Ear, Neck, and Larynx

Apply knowledge of the structure and function of the oral cavity, nose, nasal cavity and sinuses, ear, neck, and larynx to an understanding of the pathogenesis and clinicopathologic features associated with these disorders.  

*Objective HN3.1: Teeth and Gums—Non-neoplastic Disorders*. Outline the pathogenesis and clinicopathologic features of common disorders affecting the teeth and their supporting structures.  

*Objective HN3.2: Oral Cavity and Pharynx—Local and Systemic Disorders—Non-neoplastic Disorders*. Describe the pathogenesis and clinicopathologic features of local and systemic disorders affecting the oral cavity and oropharynx such as aphthous ulcers, pharyngitis, tonsillitis, oral candidiasis, Herpes simplex infections, and hairy leukoplakia.  

*Objective HN3.3: Oral Cavity, Pharynx and Teeth—Developmental Disorders*. Explain the pathogenesis and clinicopathologic features of congenital disorders involving the lip, oral cavity, and oropharynx.  

*Objective HN3.4: Nose, Nasal Cavity and Sinuses—Local and Systemic Disorders*. Discuss the pathogenesis and clinicopathologic features of local and systemic disorders (infectious and non-infectious) affecting the nose, nasal cavity, and sinuses such as rhinitis, polyps, and sinusitis.  

*Objective HN3.5: Larynx and Lower Airways—Non-neoplastic Disorders*. Compare and contrast the pathogenesis and clinicopathologic features of local and systemic disorders affecting the larynx and lower airways (epiglottitis, laryngitis, vocal cord nodules and polyps, and squamous papilloma).  

*Objective HN3.6: Larynx and Lower Airways—Developmental Disorders*. Discuss the pathogenesis and clinicopathologic features of congenital disorders affecting the larynx and lower airways.  

*Objective HN3.7: Nose, Nasal Cavity and Sinuses—Developmental Disorders*. Explain the pathogenesis and clinicopathologic features of congenital disorders affecting the nose, sinuses, and nasopharynx.  

*Objective HN3.8: Neck—Developmental Disorders*. Compare and contrast the pathogenesis and clinicopathologic features of congenital disorders affecting the neck such as branchial cleft cyst and thyroglossal duct cyst.  

*Objective HN3.9: Ear—Non-neoplastic Disorders*. Discuss the pathogenesis and clinicopathologic features of inflammatory and infectious disorders affecting the ear such as otitis media, otitis externa, cholesteatoma, and otosclerosis.  

*Objective HN3.10: Ear—Developmental Disorders*. Describe the pathogenesis and clinicopathologic features of congenital disorders affecting the ear.  

*Objective HN3.11: Larynx and Lower Airways—Non-neoplastic Disorders*. Compare and contrast the pathogenesis and clinicopathologic features of local and systemic disorders affecting the larynx and lower airways such as epiglottitis, laryngitis, vocal cord nodules and polyps, and squamous papilloma.  

*Objective HN3.12: Larynx and Lower Airways—Developmental Disorders*. Discuss the pathogenesis and clinicopathologic features of congenital disorders affecting the larynx and lower airways.

### Topic: **Gastrointestinal Tract (GT)**

Gastrointestinal (GI) tract disorders resulting from abnormal development, genetic mutations, immune disorders, infections, neoplasms, and intrinsic disease as they relate to abnormalities of the esophagus, stomach, and intestine are enumerated.

#### Learning Goal 1: Developmental Disorders of the Gut

Apply knowledge of the embryology of the foregut, midgut, and hindgut to summarize the morphological features and clinical presentation of developmental anomalies.  

*Objective GT1.1: Congenital Disorders of the Gut*. Discuss the clinicopathological features of tracheoesophageal fistula, pyloric stenosis, intestinal atresia, Meckel diverticulum, anal atresia, and Hirschsprung disease.

#### Learning Goal 2: Vascular Disorders of the Gut

Apply knowledge of the gross anatomy of the GI tract and hemodynamic principles to discuss vascular disorders of the gut.  

*Objective GT2.1: Ischemic Disorders of the Gut*. Explain the pathogenesis and clinicopathological features for common disorders of the GI tract that arise from hypoxia or ischemia.  

*Objective GT2.2: Necrotizing Enterocolitis*. Discuss the pathophysiology and clinicopathologic features of necrotizing enterocolitis.  

*Objective GT 2.3: Gastrointestinal Bleeding*. Explain the pathogenesis and clinicopathological features and outline the underlying disorders associated with hematemesis, hematochezia, and melena.  

#### Learning Goal 3: GI Neoplasia

Apply knowledge of neoplasia to explain the clinical presentation, inheritance risk, biologic behavior, morphologic appearance, classification, diagnosis, prognosis, and targeted therapy of gastrointestinal neoplasms.  

*Objective GT3.1: Precursors to Gastrointestinal Neoplasia*. Discuss the precursor lesions, risk factors, and hereditary cancer syndromes that lead to GI neoplasia.  

*Objective GT3.2: Molecular Basis of Gastrointestinal Neoplasms*. Summarize the molecular basis and clinicopathologic features, local and systemic, for esophageal cancer, gastric cancer, GI lymphoma, gastrointestinal stromal tumor, colon cancer, and anal cancer.  

*Objective GT3.3: Esophageal Carcinoma*. Compare and contrast adenocarcinomas and squamous cell carcinoma of the esophagus with respect to location, pathologic features, and major risk factors.  

*Objective GT3.4: Colon Carcinoma*. Discuss the two most important prognostic factors for colon cancer and explain why they are most important.  

*Objective GT3.5: Colonic Polyps*. Compare and contrast the different types of colonic polyps and their risk of developing cancer.  

*Objective GT3.6: (Formerly GT4.1): Right- and Left-Sided Colon Carcinoma*. Distinguish between carcinomas arising in the left and right colon in terms of symptoms and morphology.  

*Objective GT3.7: (Formerly GT4.2): Staging of Colon Carcinoma*. Describe how colon cancers are staged and list the common sites of metastases.  

*Objective GT3.8: Esophageal Neoplasia*. Discuss the etiology, pathogenesis, and clinicopathologic features of benign, mesenchymal, and other non-carcinomatous neoplasms involving the esophagus.  

*Objective GT3.9: Gastric Polyps and Masses*. Describe the etiology, pathogenesis, and clinicopathologic features of common causes of gastric polyps and masses.  

*Objective GT3.10: Anal Neoplasia*. Explain the etiology, pathogenesis, and clinicopathologic features associated with anal neoplasia.  

*Objective GT3.11: Peritoneal Cavity*. Discuss the etiology, pathogenesis, and clinicopathologic features of neoplasms affecting the peritoneal cavity.  

*Learning Goal 4: Features of Gastrointestinal Neoplasms is retired and combined into Learning Goal 3*

#### Learning Goal 5: Immune-Related Disorders of the GI Tract

Apply knowledge of immune system dysregulation and the normal structure and function of the intestines to discuss specific immune-related disorders affecting the GI tract.  

*Objective GT5.1: Inflammatory Bowel Disease*. Compare and contrast the pathophysiology and clinicopathological features of inflammatory bowel disease and irritable bowel syndrome.  

*Objective GT5.2: Celiac Disease*. Explain the pathophysiology of gliadin hypersensitivity (celiac disease) and the associated clinicopathologic manifestations, including potential long-term complications.  

*Objective GT5.3: Crohn Disease and Ulcerative Colitis*. Compare and contrast Crohn disease and ulcerative colitis, including their pathogenesis, distribution of disease, morphologic features, and complications.  

*Objective GT5.4: Autoimmune Disorders*. Compare and contrast the pathophysiology and clinicopathologic features of common autoimmune disorders affecting the GI tract and their complications.  

#### Learning Goal 6: Malabsorption

Apply knowledge of gastrointestinal anatomy and physiology to summarize the clinicopathologic features and diagnostic criteria of disorders presenting with malabsorption.  

*Objective GT6.1: Systemic Disorders with Malabsorption*. Compare and contrast the pathogenesis and clinicopathologic features of systemic disorders leading to malabsorption.  

*Objective GT6.2: Pancreaticobiliary Causes of Malabsorption*. Outline disorders of the pancreas and bile acid metabolism and discuss how they lead to malabsorption.  

*Objective GT6.3: Inflammatory Causes of Malabsorption*. Explain how celiac disease, non-celiac sprue, gastroenteritis, and inflammatory bowel disease lead to malabsorption.  

#### Learning Goal 7: GI Infections

Apply knowledge of common pathogens and principles of immunity to describe the morphological features and clinical presentation of infectious diseases affecting the GI tract of immunocompetent and immunocompromised patients.  

*Objective GT7.1: GI Infections*. Compare the underlying mechanism and clinicopathologic features of GI tract involvement by common bacterial, fungal, viral, and parasitic pathogens.  

*Objective GT7.2: Helicobacter Infection*. Relate the clinicopathologic features of *Helicobacter* infection to chronic gastritis and ulcer formation.  

*Objective GT7.3: Peritonitis*. Outline the pathogenesis and clinicopathologic features of infectious and other inflammatory disorders affecting the peritoneal cavity.  

#### Learning Goal 8: Mechanical and Inflammatory Disorders of GI tract

Apply knowledge of GI anatomy and physiology and principles of inflammation and tissue injury to explain the clinicopathologic features, diagnostic criteria, and complications of disorders resulting in inflammation of the GI tract or mechanical obstruction.  

*Objective GT8.1: Dysphagia*. Describe the pathophysiology and clinicopathological features of disorders presenting with dysphagia.  

*Objective GT8.2: Bowel Obstruction*. Compare and contrast the pathophysiology of gastrointestinal disorders that present with GI obstruction, including disorders such as volvulus, hernias, adhesions, and intussusception.  

*Objective GT8.3: Diverticulosis*. Describe the pathogenesis, clinicopathologic features, and complications of diverticulosis.  

*Objective GT8.4: Appendicitis*. Describe the clinicopathologic features of acute appendicitis and discuss the clinical differential diagnosis and potential complications of this disorder.  

*Objective GT8.5: Esophagitis*. Describe the pathophysiology and clinicopathological features of disorders associated with esophagitis.  

*Objective GT8.6: Gastritis*. Describe the pathophysiology and clinicopathological features of disorders associated with acute and chronic gastritis.  

*Objective GT8.7: Peptic Ulcer Disease*. Outline the risk factors, clinicopathological features, and potential complications of peptic ulcer disease.  

*Objective GT8.8: Hypertrophic Gastropathies*. Describe the pathophysiology and clinicopathological features of disorders associated with hypertrophic gastropathy.

### Topic: Hepatobiliary **(HB)**

Hepatobiliary disorders resulting from abnormal development, genetic mutations, immune disorders, infections, toxins, neoplasms, and intrinsic disease as they relate to the liver, biliary tract, and gallbladder are enumerated.  

#### Learning Goal 1: Hepatic Infectious Disorders

Apply knowledge of pathogenic organisms infecting the liver and their transmission, natural history, pathogenesis, laboratory profiles, and histopathological patterns of injury to the prevention and diagnosis of hepatitis and other hepatic infectious disorders.  

*Objective HB1.1: Transmission of Hepatotropic Viruses*. Explain the routes of transmission of different hepatotropic viruses and how they relate to the public health measures that have been implemented to prevent their transmission.  

*Objective HB1.2: Progression of Hepatitis*. Compare and contrast the possible clinical outcomes of the major hepatotropic viruses with particular reference to the incidence of progression to chronic hepatitis and cirrhosis.  

*Objective HB1.3: Pathophysiology of Hepatitis*. Describe the pathophysiology associated with the major hepatotropic viruses and explain how this knowledge can be used to assess the presence of hepatitis and the management and prognosis of this disease.  

*Objective HB1.4: Histopathology of Hepatitis*. Explain the pathogenetic mechanisms of injury and resulting histopathological findings observed in acute and chronic viral hepatitis.  

*Objective HB1.5: Hepatic Abscess*. Describe the etiology of hepatic abscesses and the pathways that infectious agents may take to reach the liver.  

*Objective HB1.6: Cirrhosis*. Moved to C2 HB9.4.  

#### Learning Goal 2: Liver Toxins

Apply knowledge of the cellular response to injury, the pathogenic mechanisms leading to disease, and the biochemical alterations of hepatic function to explain the clinicopathologic features, prognosis, and treatment of disorders resulting from ethanol and other drugs and toxins.  

*Objective HB2.1: Steatosis*. Describe the clinicopathologic features of excessive ethanol ingestion, focusing on biochemical pathways and short- and long-term complications, and compare and contrast alcoholic with nonalcoholic fatty liver disease.  

*Objective HB2.2: Acetaminophen Toxicity*. Describe the clinicopathologic features of excessive acetaminophen ingestion focusing on biochemical pathways and short- and long-term complications.  

*Objective HB2.3: Hemochromatosis*. Moved to C2 HB6.6.  

*Objective HB2.4: Toxic and Therapeutic Injury.* Compare and contrast the effects of common toxic and therapeutic agents to the liver, including the patterns of injury, clinical consequences, and sequelae.  

#### Learning Goal 3: Hepatic Neoplasms

Apply knowledge of neoplasia to describe the clinical presentation, biologic behavior, morphologic appearance, classification, diagnosis, prognosis, and targeted therapy of hepatic neoplasms.  

*Objective HB3.1: Causes of Hepatocellular Carcinoma*. Compare and contrast, in the context of geographic location, the epidemiological importance of the known etiologic agents associated with the development of hepatocellular carcinoma and suggest public health measures that might decrease its incidence.  

*Objective HB3.2: Morphology of Hepatocellular Carcinoma*. Discuss the morphologic features that distinguish between hepatocellular carcinoma and benign neoplasms or non-neoplastic liver disease.  

*Objective HB3.3: Hepatic Adenoma*. Describe the epidemiology and clinicopathologic features of hepatic adenomas and the molecular abnormalities that predict the risk of malignant transformation.  

*Objective HB3.4: Radiology of Cirrhosis*. Retired.  

*Objective HB3.5: Metastasis to the Liver*. Describe the factors that lead to metastasis to the liver and the features of metastatic disease that distinguish it from primary neoplasms.  

*Objective HB3.6: Pediatric Liver Neoplasia*. Describe clinicopathologic features of the common benign and malignant tumors affecting the liver in children.  

*Objective HB3.7: Vascular Tumors of the Liver*. Describe the benign and malignant vascular tumors affecting the liver.  

#### Learning Goal 4: Inflammatory Hepatobiliary (HB) Disorders

Apply knowledge of the cellular response to injury, the pathogenic mechanisms leading to disease, and the biochemical alterations of hepatic function to describe the clinicopathologic features and prognosis of intrahepatic and extrahepatic biliary tract diseases.  

*Objective HB4.1: Inflammatory Disorders of the Liver*. Compare and contrast the pathogenesis and clinicopathologic features of autoimmune hepatitis, primary and secondary biliary cirrhosis, and primary sclerosing cholangitis, including associated conditions, incidence, sex predilection, etiology, clinical features, and prognosis.  

*Objective HB4.2: Congenital Disorders of the Liver*. Moved and revised. Use C2 HB6.4  

*Objective HB4.3: Cholestasis*. Compare and contrast the various hepatic cholestatic disorders, including neonatal hepatitis and biliary atresia.  

*Objective HB4.4: Pregnancy-related Liver Disorders*. Outline the pathogenesis and clinicopathologic features of hepatic disorders occurring during pregnancy.  

#### Learning Goal 5: Biliary Neoplasia

Apply knowledge of neoplasia to an understanding of the clinical presentation, biologic behavior, morphologic appearance, classification, diagnosis, and prognosis of neoplasms involving the biliary tree.  

*Objective HB5.1: Extrahepatic Biliary Carcinoma*. Describe the epidemiology, morphology, and clinical features of gallbladder and extrahepatic biliary tract carcinoma.  

*Objective HB5.2: Cholangiocarcinoma*. Describe the presenting symptoms of cholangiocarcinoma and how the symptoms relate to the location; contrast the pathologic features with hepatocellular carcinoma.  

#### Learning Goal 6: Hereditary and Congenital and Infantile Disorders of the Liver, Gallbladder, and Extrahepatic Biliary Tree

Apply knowledge of both the embryonic principles of hepatic and bile tract development and mechanisms of fibroinflammatory injury to an understanding of disorders due to maldevelopment and acquired abnormalities of the biliary tree.  

*Objective HB6.1: Congenital Hepatic Fibrosis*. Describe the inheritance, etiology, clinical and morphologic features, and prognosis of congenital hepatic fibrosis.  

*Objective HB6.2: Polycystic Liver Disease*. Describe the inheritance, etiology, clinical and morphologic features, and prognosis of polycystic liver disease.  

*Objective HB6.3: Structural Anomalies of the Biliary Tree*. Outline cystic and other benign structural lesions affecting the biliary tree.  

*Objective HB6.4: Infantile Cholestasis and Jaundice*. Compare and contrast the etiology, clinical features, and treatment of cholestasis and jaundice in infants, including physiologic jaundice of the newborn, neonatal hepatitis, and biliary atresia.  

*Objective HB6.5: Hereditary Hyperbilirubinemia*. Discuss the genetics, physiology, and manifestations of hereditary hyperbilirubinemias such as Gilbert disease.  

*Objective HB6.6: Hemochromatosis*. Discuss the clinicopathologic features of excessive iron absorption, focusing on biochemical pathways, genetic factors, and short- and long-term complications.  

*Objective HB6.7: Metabolic Disorders Affecting the Liver*. Discuss the clinicopathologic features of metabolic disorders affecting the liver (e.g., Wilson disease, alpha-1-antitrypsin deficiency, glycogen storage diseases, tyrosinemia), focusing on biochemical pathways, genetic factors, and short- and long-term complications.  

#### Learning Goal 7: Cholelithiasis and Other Gallbladder Disorders

Apply knowledge of general biochemical principles to an understanding of how gallstones develop, risk factors for their development, and their clinical presentation and complications and apply knowledge of inflammatory principles related to other manifestations of gallbladder disease.  

*Objective HB7.1: Gallstones*. Describe the risk factors, clinical features, complications, mechanisms, and composition of gallstones.  

*Objective HB7.2: Cholecystitis*. Differentiate the epidemiology, morphology, clinical features, and complications of acute and chronic cholecystitis.  

*Objective HB7.3: Empyema and Hydrops of the Gallbladder*. Differentiate the etiology, pathogenesis, morphology, and clinical features of empyema and hydrops of the gallbladder.  

#### Learning Goal 8. Circulatory Disorders Affecting Liver

Apply knowledge of the vascular anatomy and physiology and principles of coagulation to an understanding of how vascular disorders that affect the liver develop, their clinical presentation, and potential complications.  

*Objective HB8.1: Circulatory Disorders of Liver*. Discuss the etiology, clinical presentation, and consequences of altered local and systemic circulatory problems affecting the liver (e.g., chronic passive congestion, hepatic venous outflow and portal vein obstruction, and impaired blood flow to the liver).  

#### Learning Goal 9. Liver Failure

Apply knowledge of anatomy and hepatobiliary physiology to an understanding of the clinicopathologic features and complications resulting from acute and chronic disorders of liver function.  

*Objective HB9.1: Hepatic Failure*. Discuss the pathogenesis, general clinicopathologic features, and potential complications and outcomes of acute and chronic liver failure with examples of etiologies that may cause either or both.  

*Objective HB9.2: Portal Hypertension*. Outline the common causes, basic underlying mechanisms, and consequences of portal hypertension.  

*Objective HB9.3: Jaundice*. Describe the pathogenesis and clinicopathologic features of common disorders leading to jaundice.  

*Objective HB9.4 (Formerly HB1.6): Cirrhosis*. Classify the types of cirrhosis in terms of etiology, pathogenesis, and morphologic pattern (gross and microscopic).

### Topic: **Pancreas (P)**

Pancreatic disorders resulting from abnormal development, genetic mutations, immune mechanisms, infections, neoplasms, and intrinsic disease as they relate to exocrine pancreatic abnormalities are enumerated.  

#### Learning Goal 1: Non-neoplastic Disorders of the Exocrine Pancreas

Apply knowledge of the structure and function of the pancreas to an understanding of the clinicopathologic features and diagnostic criteria of disorders resulting from cellular injury to the exocrine pancreas.  

*Objective P1.1: Pancreatitis*. Compare and contrast acute and chronic pancreatitis in terms of etiology, pathogenesis, morphologic features, and complications.  

*Objective P1.2: Genetic Disorders of the Pancreas*. Describe, with examples, genetic disorders that affect the function of the exocrine pancreas.  

#### Learning Goal 2: Pancreatic Neoplasia

Apply knowledge of the molecular basis of neoplasia to an understanding of the clinical presentation, biologic behavior, morphologic appearance, classification, diagnosis, prognosis, and targeted therapy of pancreatic neoplasms.  

*Objective P2.1: Neoplasia of the Pancreas*. Describe the major types of neoplasms affecting the exocrine pancreas.  

*Objective P2.2: Clinical Features of Pancreatic Adenocarcinoma*. Explain how the location of a pancreatic neoplasm determines its presenting symptoms and discuss the risk factors for pancreatic adenocarcinoma.  

*Objective P2.3: Endocrine Neoplasms of the Pancreas*. Retired. Use C2 EN5.5.  

*Objective P2.4: Pathologic Features of Pancreatic Adenocarcinoma*. Describe the gross and histologic features of invasive ductal adenocarcinoma of the pancreas, its precursor lesions, risk factors, and common molecular alterations.  

*Objective P2.5: Cystic Neoplasms of the Pancreas*. Compare and contrast the clinical and pathologic features of the various types of pancreatic cystic neoplasms and pseudocysts.

### Topic: Kidney **(UTK)**

Kidney disorders resulting from abnormal development, genetic mutations, immune mechanisms, infections, neoplasms, and intrinsic disease as they relate to renal abnormalities are enumerated.  

#### Learning Goal 1: Renal Neoplasia

Apply knowledge of neoplasia to explain the clinical presentation, biologic behavior, morphologic appearance, classification, diagnosis, and prognosis of renal neoplasms.  

*Objective UTK1.1: Renal Cell Carcinoma*. Compare and contrast the 3 major types of renal cell carcinoma (clear cell, papillary, and chromophobe) in terms of clinical presentation, diagnostic morphological features, and molecular pathogenesis.  

*Objective UTK1.2: Urothelial and Renal Cell Carcinoma*. Compare and contrast pelvic urothelial malignancies with renal cell carcinomas in relation to risk factors, gross and microscopic appearance, and biological behavior.  

*Objective UTK1.3: Grading and Staging of Renal Carcinoma*. Describe how renal cell carcinoma is graded and staged and discuss the factors that determine prognosis.  

*Objective UTK1.4: Wilms Tumor*. Describe the clinical and pathologic features and molecular basis for Wilms tumor and list the histologic features that are important to recognize in determining prognosis and the etiology of Wilms tumor as part of different syndromes.  

#### Learning Goal 2: Structure and Function of the Kidney

Apply knowledge of kidney structure and function to summarize how acquired and hereditary abnormalities of the parenchyma cause acute and/or chronic renal dysfunction.  

*Objective UTK2.1: Tubulointerstitial Diseases*. Describe the clinicopathological features and pathogenesis of tubulointerstitial diseases and discuss how their pathogenesis relates to treatment and outcomes.  

*Objective UTK2.2: Nephritis*. Compare and contrast acute pyelonephritis and drug-induced interstitial nephritis in terms of pathogenesis, clinical presentation, histopathological appearance, and treatment.  

*Objective UTK2.3: Acute Tubular Injury*. Compare and contrast ischemic and nephrotoxic forms of acute tubular injury, including typical clinical contexts, pathogenesis of renal failure, microscopic appearance, and expected outcome.  

*Objective UTK2.4: Chronic Renal Inflammatory Injury*. Compare and contrast chronic pyelonephritis and reflux nephropathy, including the organisms commonly associated with each.  

*Objective UTK2.5: Renal Cysts.* Compare and contrast simple and acquired cystic lesions of renal parenchyma.  

#### Learning Goal 3: Renal Vascular Dysfunction

Compare and contrast the common causes of renal vascular dysfunction in terms of size and types of vessels involved, characteristic gross and microscopic morphology, pathogenesis, and clinical presentation.  

*Objective UTK3.1: Renal Artery Occlusion*. Compare thrombotic and embolic causes of renal arterial occlusions in terms of underlying pathogenesis, gross and microscopic pathological features, and clinical presentation.  

*Objective UTK3.2: Renal Changes in Hypertension*. Discuss how the pathogenesis of hypertension leads to structural changes in the renal vasculature and how the characteristic pathological vascular lesions of the kidney seen in hypertension cause renal dysfunction.  

*Objective UTK3.3: HUS and TTP*. Compare and contrast typical hemolytic uremic syndrome (HUS), atypical HUS, and thrombotic thrombocytopenic purpura (TTP) in terms of clinical presentation, renal histopathology, pathogenesis, and prognosis.  

*Objective UTK3.4: Renal Artery Stenosis*. Describe the clinical and pathologic features associated with renal artery atherosclerosis and fibromuscular dysplasia.  

#### Learning Goal 4: Congenital Disorders of the Kidney

Apply knowledge of the embryologic principles of kidney and lower urinary tract development to explain developmental anomalies.  

*Objective UTK4.1: Inherited Renal Disorders*. Compare autosomal dominant and autosomal recessive polycystic kidney disease in terms of pathological anatomy, molecular pathogenesis, and clinical presentation.  

*Objective UTK4.2: Developmental Urinary System Disorders*. List common malformations of the urinary system and their clinical consequences.  

#### Learning Goal 5: Glomerular Disorders

Apply knowledge of the structure and function of the kidney to describe the pathogenetic mechanisms, diagnostic criteria, and clinicopathologic features of glomerular diseases presenting with asymptomatic proteinuria, nephrotic syndrome, and nephritic syndrome.  

*Objective UTK5.1: Nephritic Syndrome*. Describe the clinical features of nephritic syndrome and the proliferative and proinflammatory pathologies of conditions with this presentation.  

*Objective UTK5.2: Nephrotic Syndrome*. Describe the pathophysiology, clinical, and morphologic features of nephrotic syndrome.  

*Objective UTK5.3: Immune-mediated Renal Disease*. Compare and contrast the mechanisms of immune complex and antibody-mediated glomerulonephritis.  

*Objective UTK5.4: Diabetic Nephropathy*. Describe the pathogenesis of diabetic nephropathy and the associated clinicopathologic features.  

*Objective UTK5.5: Dysproteinemic Nephropathies*. Describe the pathogenesis of the nephropathies associated with dysproteinemia.  

*Objective UTK5.6: Lupus Nephritis*. Describe the spectrum of pathologic changes in the kidney associated with systemic lupus erythematosus.  

*Objective UTK5.7: Isolated Glomerular Hematuria*. Compare and contrast the glomerular disorders that primarily manifest as hematuria, including the morphologic and clinical findings, to include Alport syndrome, thin basement membrane nephropathy, and IgA nephropathy.  

*Objective UTK5.8: Nephrotic Glomerulopathies*. Compare and contrast the glomerular diseases that present as nephrotic syndrome.

### Topic: Bladder **(UTB)**

Bladder disorders resulting from abnormal development, genetic mutations, infections, neoplasms, obstruction, and intrinsic disease as they relate to bladder/urinary tract abnormalities are enumerated.  

#### Learning Goal 1: Bladder Neoplasia

Apply knowledge of the molecular basis of neoplasia to describe the clinical presentation, biologic behavior, morphologic appearance, classification, diagnosis, and prognosis of bladder neoplasms.  

*Objective UTB1.1: Urothelial Carcinoma*. Compare and contrast the different precursor lesions of urothelial carcinoma in terms of architecture, cytologic features, molecular–genetic changes, and propensity for invasion/progression.  

*Objective UTB1.2: Risk Factors for Urothelial Carcinoma*. Relate the risk factors for urothelial carcinoma to general principles of carcinogenesis.  

*Objective UTB1.3: Diagnosis and Surveillance of Urothelial Carcinoma*. Describe the typical clinical presentation of urothelial carcinoma and the advantages and limitations of urine cytology in diagnosis and surveillance of urothelial carcinoma.  

*Objective UTB1.4: Staging of Bladder Cancer*. Relate stage of bladder cancer to prognosis and therapy, including the role of BCG, in the treatment of low-stage tumors.  

*Objective UTB1.5: Squamous Cell Carcinoma and Other Neoplasms of the Bladder*. Discuss the risk factors associated with the development of squamous cell carcinoma of the bladder.  

#### Learning Goal 2: Bladder Infection

Apply knowledge of innate and adaptive immunity and pathogenic organisms infecting the bladder and their transmission to explain the natural history, pathogenesis, diagnosis, histopathological features, and prevention of cystitis.  

*Objective UTB2.1: Acute Cystitis*. Discuss the typical clinical symptomatology of acute cystitis and the organisms commonly causing this disorder.  

*Objective UTB2.2: Non-infectious Cystitis*. Describe the most common non-infectious causes of cystitis.  

*Objective UTB2.3: Cystitis Associated with Bladder Mass*. Describe examples in which cystitis may result in mass lesions or morphologic lesions of the urinary bladder and describe the pathogenesis of the process.  

#### Learning Goal 3: Urinary Obstruction

Apply knowledge of the anatomy and physiology of the kidney to describe how disorders may lead to obstruction of urinary outflow.  

*Objective UTB3.1: Bladder Diverticula*. Describe the pathogenesis of bladder diverticula, including congenital and acquired, and their potential role in infection, lithiasis, obstruction, and occult carcinoma.  

*Objective UTB3.2: Urinary Stones*. List the different chemical types of urolithiasis and explain the pathophysiologic mechanisms related to the development and therapy/prevention of urinary stones.  

*Objective UTB3.3: Causes of Urinary Obstruction*. Explain and give specific examples of several causes of urinary obstruction.

### Topic: Male **Reproductive—Penis (MRP)**

Penile disorders resulting from congenital anomalies, infections, and neoplastic disease as they relate to the penis are enumerated.  

#### Learning Goal 1: Non-neoplastic Disorders of the Penis

Apply knowledge of urinary tract embryology, inflammatory mechanisms, and infectious agents to summarize the epidemiology, clinicopathological features, and treatment strategies for non-neoplastic disorders of the penis.  

*Objective MRP1.1: Penile Congenital Anomalies*. Discuss the morphology, consequences, and management of phimosis, hypospadias, and epispadias.  

*Objective MRP1.2: Penile Infectious Disorders*. List the common causes of penile infections, both sexually transmitted and acquired through other routes.  

*Objective MRP1.3: Peyronie Disease*. Describe the underlying abnormality causing Peyronie disease, the clinical consequences, and its treatment.  

#### Learning Goal 2: Neoplastic Disorders of the Penis

Apply knowledge of neoplasia to describe the clinical presentation, biologic behavior, morphologic appearance, classification, diagnosis, prognosis, and therapy of penile neoplasms.  

*Objective MRP2.1: Penile Squamous Neoplasia*. Explain the spectrum of squamous neoplasia affecting the penis, ranging from condyloma acuminata to invasive squamous cell carcinoma, describing the risk factors, pathogenesis, morphologic features, complications, and treatment strategies.

### Topic: Male Reproductive—Prostate **(MP)**

Prostate disorders resulting from genetic mutations, infections, neoplasms, and intrinsic disease as they relate to prostate abnormalities are enumerated.  

#### Learning Goal 1: Prostate Neoplasia

Apply knowledge of the molecular and cellular origins of prostate cancers, specifically adenocarcinoma, to summarize the epidemiology, clinicopathological features, natural history, and treatment strategies for this disease.  

*Objective MP1.1: Prostate Adenocarcinoma*. Outline the cellular phenotype of the typical prostate adenocarcinoma cell and describe its molecular and immunohistochemical characteristics.  

*Objective MP1.2: Histopathologic Criteria for Prostate Adenocarcinoma*. Define the histopathological diagnostic criteria for the diagnosis of prostatic adenocarcinoma.  

*Objective MP1.3: Epidemiology of Prostate Adenocarcinoma*. Explain the epidemiology of prostate cancer with respect to age and family history.  

*Objective MP1.4: “Histological” versus “Clinically Significant” Prostatic Adenocarcinoma*. Compare and contrast the significance of “histological” prostatic adenocarcinoma versus a “clinically significant” adenocarcinoma.  

#### Learning Goal 2: Non-neoplastic Disorders of the Prostate

Apply knowledge of the molecular and cellular origins of non-neoplastic disorders of the prostate, specifically prostatitis and nodular hyperplasia, to explain the epidemiology, clinicopathological features, natural history, and treatment strategy for these diseases.  

*Objective MP2.1: Nodular Hyperplasia*. Explain the molecular and hormonal origins of prostatic nodular hyperplasia, the area of the gland affected, the natural history of the disease, various treatment strategies, and anticipated outcomes of treatment.  

*Objective MP2.2: Prostatitis*. Describe the pathophysiologic basis for inflammatory conditions affecting the prostate, including the causative organisms.

### Topic: Male Reproductive—Testes **(MT)**

Testicular disorders resulting from abnormal development, neoplasms, infections, and intrinsic disease as they relate to testicular abnormalities are enumerated.  

#### Learning Goal 1: Non-neoplastic Disorders of the Testes and Epididymis

Apply knowledge of the molecular and cellular origins of non-neoplastic disorders of the testis to explain the epidemiology, clinicopathological features, natural history, and treatment strategy for these diseases.  

*Objective MT1.1: Cryptorchidism*. Name the structure through which the testes descend during fetal development and what is brought with the testes in the descent. Describe the complications observed for failure of the testes to descend (cryptorchidism).  

*Objective MT1.2: Testicular Torsion*. Describe the clinical features and pathologic findings in the testis that occur due to torsion of the spermatic cord.  

*Objective MT1.3: Orchitis and Epididymitis*. Discuss several inflammatory conditions affecting the testis and epididymis and the clinicopathologic features associated with each.  

*Objective MT1.4: Scrotal Swelling*. Compare and contrast the pathogenesis and clinical consequences of disorders related to the tunica vaginalis.  

#### Learning Goal 2: Testicular Neoplasia

Apply knowledge of the molecular and cellular origins of the common types of testicular cancer to explain the epidemiology, clinicopathological features, natural history, and treatment strategies for this disease.  

*Objective MT2.1: Tumors of the Testis*. Describe the most important risk factors, genetic associations, and molecular basis for the development of testicular tumors and outline the clinicopathologic features for the different morphologic patterns seen.  

*Objective MT2.2: Diagnosis of the Testicular Mass*. Retired.

### Topic: **Breast (BR)**

Breast disorders resulting from abnormal development, genetic mutations, immune mechanisms, infections, neoplasms, and intrinsic disease as they relate to breast abnormalities are enumerated.  

#### Learning Goal 1: Non-neoplastic Disorders of the Breast

Apply knowledge of embryology, cellular responses to injury, neoplasia, and biologic and molecular alterations to describe the clinical presentation, inheritance risk, biologic behavior, morphologic appearance, classification, diagnosis, prognosis, and therapy of non-neoplastic disorders of the breast.  

*Objective BR1.1: Clinical Presentation of Breast Lesions*. Identify the most frequently diagnosed breast lesions by age of the patient, based on the most common clinical presentations in men versus women.  

*Objective BR1.2: Silicone Breast Implants*. Discuss silicone breast implants in terms of the morphologic changes in the adjacent breast and the risk of subsequent autoimmune disease and cancer.  

*Objective BR1.3: Inflammatory Breast Conditions*. Compare and contrast inflammatory breast conditions in terms of etiology, pathogenesis, morphology, and clinical features.  

*Objective BR1.4: Proliferative and Nonproliferative Breast Changes*. Discuss the clinical significance of proliferative and nonproliferative breast changes, with and without atypia, and describe how each of these changes and the family history affects the subsequent risk of developing breast cancer.  

*Objective BR1.5: Developmental Breast Disorders*. Describe the clinical features of congenital and developmental disorders of the breast.  

#### Learning Goal 2: Breast Neoplasms

Apply knowledge of the molecular basis of neoplasia to describe the clinical presentation, biologic behavior, morphologic appearance, classification, diagnosis, prognosis, and targeted therapy of breast neoplasms.  

*Objective BR2.1: Fibroadenoma and Phyllodes Tumor*. Compare and contrast fibroadenoma and phyllodes tumor in terms of clinical features, morphologic findings, and prognosis.  

*Objective BR2.2: Precursors to Breast Carcinoma*. Describe the proposed precursor-carcinoma sequence in breast cancer and name the characteristic morphologic changes.  

*Objective BR2.3: Breast Carcinoma in Situ*. Compare and contrast ductal carcinoma in situ (DCIS) and lobular carcinoma in situ (LCIS) in terms of incidence, clinical presentation, morphology, biomarker expression, pattern of spread, natural history, treatment, and prognosis.  

*Objective BR2.4: Breast Cancer Susceptibility Genes*. For the most common breast cancer susceptibility genes, describe the normal function of the gene product, incidence of gene mutation, reasons for its association with cancer, percentage of hereditary breast cancer, and risk of breast cancer by age 70.  

*Objective BR2.5: Gene Expression in Breast Cancer*. Explain the major molecular classes of invasive ductal carcinoma of the breast identified by gene expression profiling and describe how each correlates with prognosis and response to therapy.  

*Objective BR2.6: Categories of Breast Cancer*. Construct a table to compare and contrast invasive ductal carcinoma, invasive lobular carcinoma, medullary carcinoma, colloid (mucinous) carcinoma, tubular carcinoma, and metaplastic carcinoma of the breast in terms of incidence, age predilection, etiology, pathogenesis, clinical presentation, gross and microscopic morphology, grade, molecular classification, patterns of spread, clinical course, prognostic indicators, treatment options, and survival rates, indicating which are more common in men versus women.  

*Objective BR2.7: Factors Affecting Response and Prognosis of Breast Cancer*. Explain the prognosis and likelihood of recurrence and response to therapy for patients having breast cancer based on knowledge of molecular classification and/or gene expression profiling, morphologic classification, grade, prognostic marker studies, and other predictive factors.

### Topic: Female Reproductive—Uterus, Cervix, and Vagina **(FU)**

Uterine disorders resulting from abnormal development, genetic mutations, infections, neoplasms, and intrinsic disease as they relate to uterine abnormalities are enumerated.  

#### Learning Goal 1: Uterine Neoplasia

Apply knowledge of the molecular basis of neoplasia to describe the clinical presentation, biologic behavior, morphologic appearance, classification, diagnosis, prognosis, and targeted therapy of uterine neoplasms.  

*Objective FU1.1: Clinical Features of Uterine Neoplasms*. Compare and contrast common benign and malignant uterine neoplasms, including important clinicopathological features related to treatment and prognosis.  

*Objective FU1.2: Endometrial Carcinoma*. Compare and contrast the precursors, clinical setting, risk factors, pathologic findings, and prognosis for type I and type II carcinomas of the endometrium.  

*Objective FU1.3: Hereditary Colorectal Cancer and Endometrial Carcinoma*. Discuss the relationship of endometrial carcinoma to hereditary non-polyposis colorectal carcinoma.  

*Objective FU1.4: Smooth Muscle Tumors of the Uterus*. Discuss the natural history, clinical presentation, and management of benign smooth muscle tumors of the uterus and the risk for malignant transformation.  

*Objective FU1.5: Uterine Carcinosarcoma*. Discuss the clinicopathologic features and molecular alterations associated with carcinosarcoma of the uterus (malignant mixed Müllerian tumor).  

#### Learning Goal 2: Non-neoplastic Uterine Disorders

Apply knowledge of uterine physiology, endocrinology, and anatomy to compare and contrast the clinical presentation and pathology of common non-neoplastic uterine disorders.  

*Objective FU2.1: Endometrial Hyperplasia*. Define endometrial hyperplasia and discuss its etiology, classification, and prognosis.  

*Objective FU2.2: Menstrual Cycle*. Identify the phases of the menstrual cycle and the major hormonal changes that occur, comparing normal menstruation to common causes of abnormal bleeding in adolescents, perimenopausal, and postmenopausal women.  

*Objective FU2.3: Uterine Adenomyosis*. Compare and contrast the pathology of adenomyosis with endometriosis.  

*Objective FU2.4: Abnormal Uterine Bleeding*. Discuss the causes of abnormal uterine bleeding, including hormonal disturbances, anovulatory cycle, endometriosis, acute and chronic endometritis, and endometrial polyps.  

#### Learning Goal 3: Cervical Neoplasia

Apply knowledge of molecular and virus-induced principles of neoplasia to describe the detection, clinical presentation, pathogenesis, morphologic appearance, and prevention of cervical neoplasms.  

*Objective FU3.1: Clinical Features of Cervical Dysplasia and Neoplasms*. Discuss the common human papillomavirus types that affect the cervix and discuss the pathogenesis of cervical dysplasia and neoplasia, and cervical screening methods and prevention.  

*Objective FU3.2: Pathologic Features of Cervical Dysplasia and Neoplasms.* Discribe the morphologic features of the spectrum of cervical dysplasia and neoplasia derived from both cytologic and tissue specimens and clinical outcomes associated with each.  

#### Learning Goal 4: Female Genital Tract

Apply knowledge of embryology, physiology, anatomy, infectious diseases, and neoplasia to compare and contrast the clinical presentation and pathology of common female genital tract disorders.  

*Objective FU4.1: Clinical Features of Pelvic Infections*. Discuss common pelvic infections, including those affecting the vulva, vagina, cervix, and fallopian tubes and describe the pathogenesis of pelvic inflammatory disease, common organisms involved, and its complications.  

*Objective FU4.2: Developmental Disorders of the Female Genital Tract*. List several congenital and developmental anomalies of the vagina, cervix, and uterus with a discussion of clinical and morphologic findings and relevant risk factors.  

*Objective FU4.3: Vulvar Epithelial Lesions*. List the differential diagnosis for benign and neoplastic lesions of the vulvar epithelium as well as the clinical and pathologic features of each, including lichen sclerosus, squamous cell hyperplasia, vulvar intraepithelial neoplasia, vulvar carcinoma, and extramammary Paget disease.  

*Objective FU4.4: Vaginal Rhabdomyosarcoma*. Describe the clinicopathologic features of vaginal embryonal rhabdomyosarcoma (sarcoma botryoides).

### Topic: Female Reproductive—Ovary **(FO)**

Ovarian disorders resulting from abnormal development, genetic mutations, infections, immune mechanisms, neoplasms, and intrinsic disease as they relate to the ovary are enumerated.  

#### Learning Goal 1: Ovarian Neoplasia

Apply knowledge of the molecular basis of neoplasia to describe the clinical presentation, biologic behavior, morphologic appearance, classification, diagnosis, prognosis, and targeted therapy of ovarian neoplasms  

*Objective FO1.1: Ovarian Development*. Describe the embryologic development and the histologic components of the ovary, including surface Müllerian epithelium, germ cells, and sex cord-stromal cells.  

*Objective FO1.2: Causes of Ovarian Neoplasms*. Describe the risk factors, genetic associations, and molecular basis, including hereditary cancer syndromes, for ovarian neoplasms of epithelial, sex cord-stromal, and germ cell derivation.  

#### Learning Goal 2: Non-neoplastic Disorders of the Ovary

Apply knowledge of infectious diseases and physiology, embryology, and immunology to explain the major pathologic features of processes affecting the ovary.  

*Objective FO2.1: Infections Involving the Ovary*. Describe the pathogens that can cause ovarian disease and explain the underlying mechanisms, clinicopathologic features, and complications.  

*Objective FO2.2: Polycystic Ovary Syndrome*. Explain the pathophysiologic basis of polycystic ovary syndrome and clinical consequences of the disorder.  

*Objective FO2.3: Immune Diseases of the Ovary*. Explain the mechanism(s) by which the dysregulation of the immune system gives rise to ovarian disease and describe the pathology observed.  

*Objective FO2.4: Menopause*. Describe the clinicopathologic features of menopause and the basis for treatment.

### Topic: Female Reproductive—Disorders of Pregnancy **(FDP)**

Pregnancy disorders resulting from abnormal implantation, genetic mutations, hemodynamic disturbances, immune mechanisms, infections, and intrinsic disease as they relate to gestational disease abnormalities are enumerated.  

#### Learning Goal 1: Disorders of Pregnancy

Apply knowledge of embryology, cellular responses to injury, hemodynamics, and molecular alterations to summarize the clinical presentation, morphologic appearance, classification, diagnosis, biologic behavior, and therapy for disorders of pregnancy.  

*Objective FDP1.1: Ectopic Pregnancy*. Describe risk factors, characteristic morphologic findings, potential outcomes, and the medical/surgical options for the management of ectopic pregnancy in relation to the pathogenesis and likelihood of adverse consequences.  

*Objective FDP1.2: Spontaneous Abortion*. List several fetal and maternal causes for spontaneous abortion and indicate which is the most common.  

*Objective FDP1.3: Late Pregnancy*. Describe how disorders of late pregnancy can lead to effects that threaten the mother and/or fetus.  

*Objective FDP1.4: Infections During Pregnancy*. Discuss the ascending and hematogenous infections occurring during pregnancy in terms of etiology, pathogenesis, morphology, methods of diagnosis, prognosis, and treatment.  

*Objective FDP1.5: Eclampsia*. Explain the principal pathophysiologic aberrations of the placenta and maternal circulation in preeclampsia and eclampsia; the characteristic morphologic features in the placenta, liver, kidney, and brain; and how management is affected by gestational age and severity of disease.  

*Objective FDP1.6: Gestational Trophoblastic Disease*. Explain, with specific examples, how to differentiate forms of gestational trophoblastic disease based on etiology, pathogenesis, morphologic features, clinical features, and laboratory findings, including potential consequences and/or subsequent risks, treatment, and prognosis for each.  

*Objective FDP1.7: Gestational Diabetes*. Describe the pathophysiologic effects of diabetes mellitus on the mother and fetus.

### Topic: Endocrine **(EN)**

Endocrine disorders resulting from abnormal development, genetic mutations, immune mechanisms, infections, neoplasms, and intrinsic disease as they relate to multiple endocrine organ abnormalities are enumerated.  

#### Learning Goal 1: Hyper- and Hypopituitarism

Apply knowledge of pituitary physiology to describe the pathophysiology and clinicopathologic features of disorders associated with hyperpituitarism and hypopituitarism.  

*Objective EN1.1: Anterior Pituitary*. List several causes for destruction of the anterior pituitary and the clinicopathologic features associated with each.  

*Objective EN1.2: Sheehan Syndrome*. Define Sheehan syndrome and discuss the clinicopathologic features associated with it.  

*Objective EN1.3: Posterior Pituitary*. Outline the clinicopathologic features associated with disorders affecting the posterior pituitary gland.  

#### Learning Goal 2: Hyperthyroidism and Hypothyroidism

Apply knowledge of thyroid physiology to explain the pathophysiology and clinicopathologic features of disorders associated with hyperthyroidism and hypothyroidism.  

*Objective EN2.1: Causes ofHyperthyroidism and Hypothyroidism*. Compare and contrast the causes of hyperthyroidism versus hypothyroidism.  

*Objective EN2.2: Clinical Features of Hyperthyroidism andHypothyroidism*. Compare and contrast the clinicopathologic features of hyperthyroidism versus hypothyroidism.  

#### Learning Goal 3: Autoimmune Thyroiditis

Apply knowledge of immune system dysregulation to summarize immune-related disorders of the thyroid.  

*Objective EN3.1: Graves Disease, Hashimoto Thyroiditis, and Subacute Thyroiditis*. Compare and contrast the pathophysiology and clinicopathologic features of Graves disease, Hashimoto thyroiditis, and subacute lymphocytic thyroiditis.  

*Objective EN3.2: Granulomatous Thyroiditis*. Compare and contrast immune-mediated thyroid disease with subacute granulomatous thyroiditis (de Quervain thyroiditis).  

#### Learning Goal 4: Hyper- and Hypoadrenalism

Apply knowledge of adrenal physiology to describe the pathophysiology and clinicopathologic features of disorders associated with adrenocortical hyperfunction (hyperadrenalism) and adrenocortical insufficiency.  

*Objective EN4.1: Cushing Syndrome*. Compare and contrast the causes and clinicopathologic features of hypercortisolism (Cushing syndrome), the pathophysiologic basis distinguishing between these causes, and the management of this disease.  

*Objective EN4.2: Hyperaldosteronism*. Compare and contrast the causes and clinicopathologic features of primary and secondary hyperaldosteronism.  

*Objective EN4.3: Congenital Adrenal Hyperplasia*. Outline the genetic basis and clinicopathologic features of congenital adrenal hyperplasia.  

*Objective EN4.4: Adrenocortical Insufficiency*. Compare and contrast the causes of adrenocortical insufficiency, including the pathogenesis of primary acute and chronic adrenocortical insufficiency.  

#### Learning Goal 5: Endocrine Neoplasms

Apply knowledge of the molecular basis of neoplasia to explain the clinical presentation, biologic behavior, morphologic appearance, classification, diagnosis, prognosis, and targeted therapy of endocrine neoplasms.  

*Objective EN5.1: Thyroid Neoplasms*. Compare and contrast the clinicopathologic features, pathogenesis, and prognosis of follicular adenomas, follicular carcinoma, and papillary thyroid carcinoma.  

*Objective EN5.2: Medullary Thyroid Carcinoma*. Describe the molecular basis and clinicopathologic features of medullary thyroid carcinoma.  

*Objective EN5.3: Pheochromocytoma and Paraganglioma*. Outline the clinicopathologic features of pheochromocytoma and compare and contrast the hereditary cancer syndromes associated with paragangliomas/pheochromocytomas.  

*Objective EN5.4: Pituitary Adenoma*. Explain the clinicopathologic features of pituitary adenomas, including their genetic mutations and their associated clinical syndromes.  

*Objective EN5.5: Pancreatic Neuroendocrine Tumors*. Compare and contrast the clinicopathologic features of the pancreatic neuroendocrine tumors, their genetic alterations, and complications of each.  

*Objective EN5.6: Neuroblastoma*. Describe the spectrum of clinical and pathologic features associated with the peripheral neuroblastic tumors neuroblastoma, ganglioneuroblastoma, and ganglioneuroma as well as important prognostic indicators.  

#### Learning Goal 6: Endocrine Pancreas

Apply knowledge of genetics, the structure and function of the endocrine pancreas, and biochemical principles of carbohydrate metabolism to summarize the clinicopathologic features, diagnostic criteria, and therapy of disorders resulting from the excess or decreased production of insulin and other islet cell hormones and of other hereditary conditions related to the endrocrine pancreas.  

*Objective EN6.1: Features of Diabetes Mellitus*. Compare and contrast the pathophysiology, risk factors, and clinicopathologic features of type 1 and type 2 diabetes.  

*Objective EN6.2: Complications of Diabetes Mellitus*. Outline the pathologic complications of diabetes mellitus.  

*Objective EN6.3: Multiple Endocrine Neoplasia (MEN) Syndromes*. Compare and contrast the clinicopathologic features of MEN 1 with MEN 2 and 3.  

#### Learning Goal 7: Hyperparathyroidism and Hypoparathyroidism

Apply knowledge of the structure and function of the parathyroid glands and biochemical principles of calcium, phosphate, and vitamin D metabolism to summarize the clinicopathologic features, diagnostic criteria, and therapy of disorders resulting from excess or decreased production of parathyroid hormone.  

*Objective EN7.1: Hyperparathyroidism*. Describe the causes and clinicopathologic features of primary, secondary, and tertiary hyperparathyroidism including potential effects on bone.  

*Objective EN7.2: Hypoparathyroidism*. Describe the causes and clinical features of hypoparathyroidism.

### Topic: **Skin (SK)**

Skin disorders resulting from abnormal development, genetic mutations, immune mechanisms, infections, neoplasms, and intrinsic disease as they relate to cutaneous abnormalities are enumerated.  

#### Learning Goal 1: Classification of Skin Disease

Apply knowledge of histology, cell biology, inflammation, and neoplasia to an understanding of the clinical presentation, biologic behavior, morphologic appearance, and classification of diseases of the skin.  

*Objective SK1.1: Pathophysiology of Changes in the Skin*. Describe the pathophysiologic basis for changes in the color, surface texture, swelling, temperature, and sensitivity of skin.  

*Objective SK1.2: Terminology of Clinical and Histologic Skin Changes*. Describe the gross (clinical) and microscopic appearance of skin lesions using appropriate terminology.  

#### Learning Goal 2: Barrier Breakdown of the Skin

Apply knowledge of immunology and the anatomic structure of the skin to discuss the role of skin in protecting against direct invasion of the skin and its appendages by pathogens and non-infectious agents.  

*Objective SK2.1: Non-infectious Effects on Skin Barrier*. Describe the various chemical and physical processes that result in breakdown of the cutaneous barrier and discuss the clinical consequences.  

*Objective SK2.2: Cutaneous Infections*. Describe common bacterial, viral, fungal, and parasitic agents that may cause cutaneous infections and the sites that they infect, their morphologic features, and their complications.  

*Objective SK2.3: Cutaneous Infestations*. Discuss the epidemiology, clinical features, and methods of acquiring common infestations of the skin, such as scabies, lice, and bedbugs.  

#### Learning Goal 3: Immune-related Disorders of the Skin

Apply knowledge of basic concepts in immunopathology and the key immunologic functions of components of the skin to understand the pathologic basis of disease caused by reactivity to exogenous agents versus immunologically driven disease, including those with a genetic component.  

*Objective SK3.1: Response to Exogenous Antigens*. Describe the clinical features and pathologic basis for skin changes in response to exogenous antigens including infectious organisms, drugs, chemicals, and environmental agents.  

*Objective SK3.2: Immune Diseases of the Skin*. Describe the clinical features and pathologic basis for immunologically driven skin diseases with a genetic component such as eczema, psoriasis, and vitiligo.  

*Objective SK3.3: Inflammatory Blistering Disorders*. Compare and contrast the pathogenesis, clinical findings, and pathologic features for the cutaneous blistering disorders pemphigus, bullous pemphigoid, and dermatitis herpetiformis.  

#### Learning Goal 4: Inherited Disorders of the Skin

Apply knowledge of genetics, skin structure and function, and basic principles of pathology to an understanding of non-neoplastic-inherited disorders of the skin.  

*Objective SK4.1: Inherited Diseases of the Skin*. Describe the genetic basis, manifestations, and clinical outcomes for blistering diseases and other inherited disease affecting the skin.  

#### Learning Goal 5: Skin Neoplasia

Apply knowledge of the molecular basis of neoplasia to an understanding of the clinical presentation, biologic behavior, morphologic appearance, classification, diagnosis, prognosis, and therapy of benign and malignant skin neoplasms.  

*Objective SK5.1: Benign Skin Neoplasms*. Describe the clinical presentation and histopathologic findings of benign skin growths of basal cell, squamous cell, and melanocyte origins as well as neoplasms of dermal origin.  

*Objective SK5.2: Malignant Skin Neoplasms*. Describe the clinical presentation, precursor lesions, risk factors, and hereditary cancer syndromes that lead to the following skin cancers: basal cell carcinoma, squamous cell carcinoma, and melanoma.  

*Objective SK5.3: Genetic Disorders Predisposing to Skin Cancer*. Identify the genetic disorders with a high risk of skin cancers and explain the molecular basis of that risk as well as the genomic mutations involved.  

*Objective SK5.4: Sun Exposure*. Explain the role of ultraviolet light and other environmental factors in the development of various skin cancers.  

*Objective SK5.5: Cutaneous T-Cell Lymphoma*. Describe the various clinical presentations of cutaneous T-cell lymphoma, specifically mycosis fungoides, and discuss the natural course of the disease.  

*Objective SK5.6: Cutaneous Tumors of Mast Cells and Langerhans Cells*. Compare and contrast the clinical and pathologic features of cutaneous mastocytosis and Langerhans cell histiocytosis.  

#### Learning Goal 6: Skin Appendage Disorders

Apply knowledge of histology of the skin appendages and environmental and immunologic factors to understand the pathophysiology of skin appendage disorders.  

*Objective SK6.1: Alopecia*. Compare and contrast the various clinical presentations and pathogenesis of alopecia.  

*Objective SK6.2: Skin Appendage Disorders*. Compare and contrast the clinical presentation and pathogenesis of common skin appendage disorders such as acne, panniculitis, and rosacea.

### Topic: Musculoskeletal System **(MS)**

Musculoskeletal disorders resulting from abnormal development, genetic mutations, nutritional disorders, immune mechanisms, infections, neoplasms, and intrinsic disease as they relate to muscle, bone, and soft tissue abnormalities are enumerated.  

#### Learning Goal 1: Bone Neoplasia

Apply knowledge of the molecular basis of neoplasia to describe the clinical presentation, biologic behavior, morphologic appearance, classification, diagnosis, prognosis, and targeted therapy of bone neoplasms.  

*Objective MS1.1: Categories of Bone Tumors*. Describe examples of bone forming, cartilage forming, and other common bone tumors including the clinicopathologic features, radiological findings, treatment, and prognosis of each.  

*Objective MS1.2: Bone-forming Sarcomas in Children*. Describe the most common benign and malignant bone-forming tumors in children and adolescents in terms of clinical presentation, radiologic findings, histologic features, treatment, and prognosis.  

*Objective MS1.3: Cartilage-forming Tumors*. Describe the most common benign and malignant cartilaginous tumors of bone in terms of clinical presentation, radiologic findings, histologic features, treatment, and prognosis.  

*Objective MS1.4: Metastatic Tumors*. Describe the tumors that commonly metastasize to bone, the radiologic manifestations of metastatic lesion involving bone, and the difference between osteoblastic and osteolytic metastases.  

*Objective MS1.5: Soft Tissue Tumors*. Describe the common benign and malignant soft tissue tumors, including the genetic contributions to tumor development and progression.  

#### Learning Goal 2: Non-neoplastic Disorders of the Musculoskeletal System

Apply knowledge of histology, immunology, microbiology, and biological and molecular alterations to discuss clinical presentation, biological behavior, morphological appearance, and natural history of non-neoplastic disorders of bones, joints, and skeletal muscle.  

*Objective MS2.1: Osteomalacia and Rickets*. Compare and contrast osteomalacia and rickets with respect to pathogenesis and clinicopathologic features.  

*Objective MS2.2: Osteomyelitis and Septic Arthritis*. Discuss the pathogenesis of osteomyelitis and septic arthritis, including predisposing factors, organisms involved, morphologic appearance, and complications.  

*Objective MS2.3: Osteoporosis*. Distinguish primary from secondary osteoporosis in terms of etiology, pathogenesis, and morphology.  

*Objective MS2.4: Spinal Degenerative Disease*. Describe the common degenerative diseases of the spine.  

*Objective MS2.5: Pathologic Fracture*. Compare and contrast pathologic versus non-pathologic fractures, including the potential for healing.  

*Objective MS2.6: Paget Disease*. Discuss the clinicopathologic changes of Paget disease, including the histologic phases and complications of this disorder.  

*Objective MS2.7: Arthritis*. Compare and contrast rheumatoid and osteoarthritis, including the etiology, pathogenesis, clinical features, and morphology of each.  

*Objective MS2.8: Gout*. Describe the pathogenesis of crystal-induced arthritis, including predisposing factors, clinical findings, radiology, and morphology.  

*Objective MS2.9: Inflammatory Arthritis*. Compare and contrast rheumatoid arthritis with other types of inflammatory arthritis such as juvenile idiopathic arthritis and the seronegative spondyloarthropathies.  

*Objective MS2.10: Inflammatory Myopathies*. Compare and contrast the pathogenesis, epidemiology, and clinicopathologic features of inflammatory myopathies.  

#### Learning Goal 3: Congenital Disorders of the Musculoskeletal System

Apply knowledge of histology, development, and biological and molecular alterations to discuss clinical presentation, morphological appearance, and natural history of congenital disorders of bones, joints, and skeletal muscle.  

*Objective MS3.1: Muscular Dystrophies*. Compare and contrast congenital muscular dystrophies due to dystrophin mutations to include the inheritance pattern, clinical progression and prognosis, and histologic changes to muscle.  

*Objective MS3.2: Skeletal Dysplasias*. Describe the pathogenesis, including genetic mutations, and clinical and radiologic findings associated with skeletal dysplasias to include achondroplasia, osteogenesis imperfecta, and osteopetrosis.

### Topic: Nervous System—Central Nervous System **(NSC)**

Nervous system disorders resulting from abnormal development, genetic mutations, vascular changes, immune mechanisms, infections, neoplasms, and intrinsic disease as they relate to CNS abnormalities are enumerated.  

#### Learning Goal 1: CNS Neoplasia

Apply knowledge of the pathological and molecular basis of common brain tumors to describe their clinical behavior, morphology, effects on the nervous system, and therapies.  

*Objective NSC1.1: Clinical Features of Brain Tumors*. Explain the pathophysiology underlying the signs and symptoms associated with brain tumors.  

*Objective NSC1.2: Classification of Brain Tumors*. Compare and contrast the common types of brain tumors with respect to their location, age incidence, pathogenesis, molecular basis, morphology, and prognosis.  

*Objective NSC1.3: Hereditary Tumor Syndromes*. Describe the major hereditary tumor syndromes of the CNS, the genes responsible for each syndrome, and the spectrum of tumors associated with each syndrome.  

*Objective NSC1.4: Grading of Brain Tumors*. Explain the pathophysiologic basis for grading primary brain tumors and discuss how grading relates to prognosis and governs patient management.  

*Objective NSC1.5: Complications of Brain Tumors*. Describe several complications of brain tumors and give specific examples.  

*Objective NSC1.6: Tumors Metastasizing to the CNS*. Discuss tumors that commonly metastasize to the CNS and describe the locations in which metastases may be seen.  

#### Learning Goal 2: CNS Infection

Apply knowledge of clinical features, neuroimaging studies, microbial pathogenesis, and location of lesion(s) to develop a differential diagnosis for CNS infections.  

*Objective NSC2.1: Infections of the CNS*. Compare and contrast the clinical, gross, and microscopic manifestations of common bacterial, viral, and fungal infections of the CNS.  

*Objective NSC2.2: Opportunistic Infections of the CNS*. Discuss common opportunistic infections that involve the CNS of immunocompromised individuals and describe their pathologic features.  

*Objective NSC2.3: Progressive Multifocal Leukoencephalopathy*. Describe the clinicopathologic features of progressive multifocal leukoencephalopathy and contrast them with infiltrative astrocytoma.  

*Objective NSC2.4: Suppurative Meningitis and Abscess*. Describe the gross and microscopic features of acute suppurative meningitis and brain abscess and name the organisms most commonly associated with each.  

#### Learning Goal 3: Spinal Cord Disorders

Apply knowledge of neuroanatomy, pathogenesis, and biologic behavior to develop differential diagnoses and determine appropriate therapy for disorders of the spinal cord.  

*Objective NSC3.1: Ependymoma*. Retired.  

*Objective NSC3.2: Spinal Cord Findings in Demyelinating and Neuromuscular Disorders*. Explain how examination of a spinal cord at autopsy is important for the diagnosis and classification of demyelinating and/or neuromuscular diseases.  

*Objective NSC3.3: Multiple Sclerosis*. Moved to NSC6.2.  

*Objective NSC3.4: Spinocerebellar Degeneration*. Discuss the inheritance pattern, pathogenesis, epidemiology, and clinical features of various causes of spinocerebellar degeneration.  

*Objective NSC3.5: Neoplasms Affecting the Spinal Cord*. Compare and contrast primary and secondary tumors of the spinal cord with respect to their pathogenesis, molecular basis, and prognosis.  

#### Learning Goal 4: Motor Neuron and Neuromuscular Disorders

Apply knowledge of genetic, clinical, anatomic, and neuropathologic principles to the diagnosis of neuromuscular disorders.  

*Objective NSC4.1: Amyotrophic Lateral Sclerosis*. Describe the etiology, pathogenesis, and clinical features of amyotrophic lateral sclerosis.  

*Objective NSC4.2: Mitochondrial Disorders*. Describe the etiology, pathogenesis, and clinical and histopathological features of mitochondrial diseases affecting skeletal muscle and the nervous system.  

*Objective NSC4.3: Spinal Muscular Atrophy*. Discuss the pathogenesis and clinical features of spinal muscular atrophy.  

#### Learning Goal 5: Dementia

Apply knowledge of structure and function and general pathologic concepts to describe disorders in which dementia is a component.  

*Objective NSC5.1: Amyloid and Tau in Dementia*. Define the essential underlying abnormalities of amyloid and tau proteins in the most common causes of dementia in the United States.  

*Objective NSC5.2: Abnormal Protein Processing in Neurodegenerative Disease*. Describe the primary protein processing abnormalities responsible for neurodegenerative diseases.  

*Objective NSC5.3: Alzheimer Disease*. Describe the clinical features, gross pathology, and histopathology of Alzheimer disease and name 3 regions of the brain that are usually involved.  

*Objective NSC5.4: Genes Implicated in Alzheimer Disease*. Discuss 3 genes in which mutations have been identified in patients with early onset Alzheimer disease.  

*Objective NSC5.5: Disorders of the Basal Ganglia*. Describe several diseases which involve the basal ganglia and describe how to distinguish among the diseases in terms of gross, microscopic, and clinical pathology.  

*Objective NSC5.6: Prion Diseases*. Discuss the pathogenesis of neurodegenerative disorders due to misfolded prion protein with discussion of epidemiology and clinicopathologic features of the most common types.  

*Objective NSC5.7: Other Neurodegenerative Diseases*. Describe the clinical features, gross pathology, and histopathology of other common neurodegenerative disorders such as frontotemporal dementia, Parkinson disease, and Huntington disease.  

*Objective NSC5.8: Vascular Dementia*. Outline the clinical findings of patients with vascular dementia and the causative vascular lesions.  

#### Learning Goal 6: Demyelinating Disorders

Apply knowledge of the structure and function of the brain and general immunopathology concepts to summarize disorders that result in demyelination in terms of their etiology, pathogenesis, clinical and morphologic features, natural history, and therapeutic options.  

*Objective NSC6.1: Autoimmune Mechanisms in Multiple Sclerosis*. Describe the autoimmune mechanism mediated by CD4-positive T cells that react against self-myelin antigens in multiple sclerosis and outline the clinicopathologic features of the disease.  

*Objective NSC6.2 (Formerly NSC3.3): Multiple Sclerosis*. Describe the pathogenesis, clinical presentation and course, and gross and microscopic pathologic features of multiple sclerosis.  

#### Learning Goal 7: Ischemia of the Brain

Apply knowledge of the structure and function of the brain and general pathology concepts to discuss disorders resulting from altered blood supply and hypoxia to the brain.  

*Objective NSC7.1: Stroke*. Compare and contrast the 2 major mechanisms for stroke and how treatment differs for each.  

*Objective NSC7.2: Traumatic Brain Injury*. Moved to C3 NSC8.2.  

*Objective NSC7.3: Cranial Hemorrhage*. Compare and contrast the etiologies and clinical presentations of epidural, subdural, subarachnoid, basal ganglionic, and lobar hemorrhages.  

*Objective NSC7.4: Hypertensive Hemorrhage*. Describe the mechanism of hypertensive hemorrhage and name common locations in which this occurs.  

*Objective NSC7.5: Embolic Infarction*. Describe how embolic infarcts differ from atherothrombotic infarcts in pathologic appearance and name 3 sources of emboli.  

*Objective NSC7.6: Acute Versus Chronic Brain Injury*. Compare and contrast the gross and histopathologic appearance of acute versus remote brain infarction.  

#### Learning Goal 8: CNS Response to Injury

Apply knowledge of normal histologic findings to describe changes resulting from injury to the brain and spinal cord.  

*Objective NSC8.1: CNS Response to Injury*. Describe the general morphologic changes at a cellular and tissue level in response to CNS injury, with examples to include red neurons and gliosis.  

*Objective NSC8.2 (Formerly NSC7.2): Traumatic Brain Injury*. Describe the pathologic findings seen in the most common causes of traumatic brain injury.  

*Objective NSC8.3: Chronic Traumatic Encephalopathy*. Discuss the clinicopathologic features of repetitive mechanical trauma to the CNS.  

#### Learning Goal 9: Congenital Anomalies Affecting the CNS

Apply knowledge of embryologic principles and biochemical pathways to describe the disorders associated with improper development of the brain and spinal cord or inherited metabolic disorders.  

*Objective NSC9.1: Congenital Malformations of the Brain*. Describe congenital disorders of the brain such as holoprosencephaly and Dandy-Walker malformation, including their anatomic findings, associated conditions, and clinical impact.  

*Objective NSC9.2: Neural Tube Defects*. Describe the spectrum of neural tube defects and factors that may increase the risk of development.  

*Objective NSC9.3: Congenital Metabolic Disorders Affecting the CNS*. Outline genetic metabolic disorders affecting the CNS such as neuronal storage disorders and leukodystrophies with discussion of the abnormal process, cell/structure affected, inheritance pattern, clinical findings, and prognosis.

### Topic: Nervous System—Peripheral Nervous System and Eye **(NSP)**

Nervous system disorders resulting from abnormal development, genetic mutations, vascular changes, immune mechanisms, infections, and intrinsic disease as they relate to peripheral nervous system (PNS) and ocular abnormalities are enumerated.  

#### Learning Goal 1: Peripheral Nerve Disorders

Apply knowledge of the structure and function of the peripheral nerves and general pathology and microbiology concepts to discuss peripheral nerve disorders.  

*Objective NSP1.1: Neuromuscular Junction Disorders*. Describe the clinicopathologic features of antibody-mediated disorders of the neuromuscular junction such as myasthenia gravis and Lambert-Eaton myasthenic syndrome.  

*Objective NSP1.2: Neuropathy*. Compare and contrast the clinicopathologic features of the various etiologies of neuropathy.  

*Objective NSP1.3: Neurofibromatosis*. Compare and contrast the clinicopathologic features of neurofibromatosis types 1 and 2.  

*Objective NSP1.4: Leprosy*. Compare and contrast the pathogenesis, clinicopathologic features, and complications of tuberculoid and lepromatous leprosy.  

#### Learning Goal 2: PNS Neoplasia

Apply knowledge of the pathological and molecular basis of common PNS tumors to describe their clinical behavior, effects on the nervous system, and therapies.  

*Objective NSP2.1: Hereditary Tumor Syndromes*. Describe the major hereditary tumor syndromes of the PNS, the genes responsible for each syndrome, and the spectrum of tumors, including the histology associated with each.  

*Objective NSP2.2: Tumors of the PNS*. Compare and contrast the common benign from malignant PNS tumors and outline their molecular basis and clinicopathologic features.  

#### Learning Goal 3: Ocular Disorders

Apply knowledge of the structure and function of the eye and general pathology concepts to discuss common ocular disorders.  

*Objective NSP3.1: Ocular Disorders*. Describe the clinicopathologic features of common primary and secondary disorders of the eye including macular degeneration, cataracts, glaucoma, and uveitis.  

*Objective NSP3.2: Ocular Neoplasms*. Describe the clinicopathologic features of common neoplasms of the eye including ocular melanoma and retinoblastoma.  

*Objective NSP3.3: Systemic Disorders Affecting the Eye*. Describe the pathogenesis and clinicopathologic features of ocular complications caused by systemic disorders such as hypertension and diabetes mellitus.  

*Objective NSP3.4: Eye Infections*. Compare and contrast the clinical features, etiologies, and outcomes of infections of the eye and periorbital soft tissue, to include bacterial, viral, fungal, and parasitic organisms.

### Topic: Autoimmune and Multisystem Disorders **(AIMS)**

Autoimmune and other disorders affecting multiple potential organ systems are enumerated.

#### Learning Goal 1: Autoimmune and Multisystem Disorders

Apply knowledge of principles of immunology and cell injury and the pathogenesis of autoimmunity to explain the pathogenesis, clinical manifestations, and therapeutic interventions associated with autoimmune and other disorders affecting multiple organ systems.  

*Objective AIMS1.1: Connective Tissue Disorders*. Compare and contrast systemic lupus erythematosus, rheumatoid arthritis, Sjogren syndrome, systemic sclerosis, and mixed connective tissue disease, including epidemiology, pathogenesis, clinical manifestations, organ involvement, and complications of each.  

*Objective AIMS1.2: IgG4 Disorders*. Discuss the pathogenesis, clinical manifestations, and organ system findings of IgG4-related disorders.  

*Objective AIMS1.3: Sarcoidosis*. Describe the pathogenesis, clinical manifestations, and organ system findings of sarcoidosis; compare sarcoidosis to other granulomatous disorders.  

*Objective AIMS1.4: Amyloidosis*. Compare and contrast the classification, pathogenesis, clinical manifestations, and organ system findings of various forms of amyloid deposition.  

## Competency 3

### Diagnostic Medicine and Therapeutic Pathology

Diagnosis and patient management require the student to apply their knowledge of disease mechanisms and organ system pathology to achieve efficient and effective use of clinical and anatomic laboratory testing. In addition, the student should learn the proper use of blood/blood product utilization to enable optimal diagnosis, treatment, and patient care.

There are 10 topics within this competency area. Each topic includes general learning goals and specific objectives that medical students should be able to meet upon graduation from medical school. Table 3 lists the topic areas and shows the number of goals and objectives for each.

**Table 3:** Diagnostic medicine and therapeutic pathology.

Topic	Number of Goals	Number of Objectives	Reference Code
General principles	1	11	GP
Health informatics	2	6	HI
Transfusion medicine	1	9	TM
Hematology	4	16	H
Microbiology	6	33	M
Chemistry	3	19	CHEM
Immunology	1	4	IMM
Molecular pathology, genomics, cytogenetics	5	14	GE
Autopsy	3	10	AU
Surgical pathology	4	11	SP
Cytopathology	2	8	CYP

### Topic: General Principles **(GP)**

Every physician should have an appreciation for the preanalytical, analytical, and postanalytical phases of laboratory testing. In addition, physicians need an appreciation of the statistical treatment of data that underlies test utilization. This includes, but is not limited to, the ability to choose the correct test to make a diagnosis and facilitate treatment selection and to employ the appropriate testing paradigm to monitor patients with chronic diseases, enabling optimal clinical management.  

#### Learning Goal 1: Laboratory Tests

Apply knowledge of clinical medicine, pathology, and statistics to determine the utility of a laboratory test in making a diagnosis and in monitoring chronic disease management. Explain the interpretation and limitations of clinical laboratory assays.  

*Objective GP1.1: Laboratory Errors*. Discuss, with examples of each, differences among preanalytical, analytical, and postanalytical mistakes that lead to laboratory or diagnostic errors.  

*Objective GP1.2: Sensitivity and Specificity*. Evaluate the quality of an assay in differentiating disease versus non-disease states, including graphically presenting and interpreting the data. Determine the relationship between sensitivity and specificity for an assay.  

*Objective GP1.3: Pretest Probability*. Determine the value of an assay by evaluating the impact of differing pretest probabilities such as prevalence on the positive and negative predictive value of the test. Give examples of the laboratory tests used to evaluate clinical disorders where predictive values are used to develop screening, diagnostic, prognostic, and patient management protocols.  

*Objective GP1.4: Reference Intervals*. Describe the methods and statistics used to establish reference intervals; the effect of demographics, treatments, or disease states on reference interval variability; and the difference between reference ranges and therapeutic ranges.  

*Objective GP1.5: Test Variability*. Explain the difference between technical variability and biologic variability, including how physical and chemical parameters such as sample size, hemolysis, and lipemia can affect test results. Define analytical uncertainty, precision, accuracy, and coefficient of variation and describe factors that contribute to each.  

*Objective GP1.6: Turn-around Time*. Compare and contrast appropriate uses of “stat” and “routine” test priorities with discussion of critical values and the elements of “turn-around time.” Predict which elements affect turn-around time the most.  

*Objective GP1.7: Regulatory Issues*. Explain the basic differences between Food and Drug Administration (FDA)-approved tests and laboratory-developed tests, including Clinical Laboratory Improvement Amendments waived and non-waived tests and discuss the primary regulatory issues involved in physician-office laboratories, home testing, and provider-performed microscopy.  

*Objective GP1.8: Point-of-Care Testing*. Explain how “point-of-care” (POC) testing in the physician office, multispecialty clinic, and hospital can enable better patient and population management of acute and chronic disease and why values generated using POC methods could differ from values generated in a high-throughput laboratory.  

*Objective GP1.9: Test Utilization*. Discuss a laboratory testing decision tree to make a diagnosis for a patient with a chronic disease or a protocol for monitoring such a patient, the use of appropriate testing and the impact on healthcare cost for overutilization or underutilization of laboratory testing, and the potential impact on cost.  

*Objective GP1.10: Test Economics*. Compare and contrast the cost of several common laboratory diagnostic tests and discuss the difference between the cost of a test and the charge to a patient.  

*Objective GP 1.11: Pathologists as Clinical Consultants*. Describe the roles the pathologist plays to communicate among the laboratory, patients, and members of the health care team.

### Topic: Health Informatics (HI)

Every physician needs an understanding of the role informatics plays in healthcare delivery and outcomes.  

#### Learning Goal 1: Health Informatics

Apply knowledge of clinical medicine, medical records, laboratory medicine (diagnostic tests), computer science, statistics, and data analysis to improve healthcare delivery and patient care.  

*Objective HI 1.1: Informatics*. Define informatics, contrast it with information technology, and discuss how different types of informatics are used to improve healthcare.  

*Objective HI 1.2: Data Analysis*. Outline how data acquisition and analysis, including data mining and machine learning, can improve healthcare delivery and improve patient outcomes.  

*Objective HI 1.3: Laboratory Medicine Informatics*. Describe the role laboratory information systems play in laboratory operations and in healthcare delivery.  

*Objective HI 1.4: Telepathology*. Discuss the circumstances in which telepathology can be used as a diagnostic modality and describe its advantages and limitations.  

#### Learning Goal 2: Artificial Intelligence (AI)

Apply knowledge of computer hardware, search engines, data analytics, and machine learning to the delivery of healthcare.  

*Objective HI 2.1: AI*. Define AI and discuss different types of AI tools that can be employed in patient care.  

*Objective HI 2.2: AI in Pathology*. Outline how AI can be used to enhance laboratory operations and to improve clinical decision making by providing focused diagnostic information to the clinical team.

### Topic: Transfusion Medicine (TM)

Every physician needs an understanding of transfusion medicine, which encompasses the transfusion of RBCs, platelets, and plasma products in order to correct deficiencies in patients or remove offending antibodies. Transfusions are not without risk, and knowledge of the pathophysiology of the disease and risks of transfusion are vital for physicians for optimal patient outcomes.  

#### Learning Goal 1: Concepts of Blood Transfusion

Apply knowledge of pathology, hematopoietic cell physiology, and immunology to explain concepts of blood component transfusion and the therapeutic interventions in transfusion medicine.  

*Objective TM1.1: Cellular Blood Components*. Define the cellular blood components and blood component substitutes available for clinical use, the evidence-based indications and dosing for transfusion of these components, and how the efficacy of transfusion may be monitored.  

*Objective TM1.2: Transfusion Reactions*. Compare and contrast the pathophysiology, presentations, prophylaxis, and management of the different types of transfusion reactions.  

*Objective TM1.3: Infectious Risks*. Discuss infectious disease risks of transfusion.  

*Objective TM1.4: HLA*. Explain the HLA system and its role in both transfusion and transplantation.  

*Objective TM1.5: Apheresis*. Explain the clinical role of therapeutic apheresis in the management of the following disorders: sickle cell anemia, TTP, acute and chronic inflammatory demyelinating polyneuropathy, myasthenia gravis, anti-glomerular basement membrane disease, organ transplantation, plasma cell dyscrasias, leukemia, and lymphoma.  

*Objective TM1.6: Paternity Testing*. Retired  

*Objective TM 1.7: Blood Group Antigens*. Describe the major antigens present on RBCs and discuss their clinical relevance using the examples of specific clinical situations in which laboratory diagnosis is needed.  

*Objective TM1.8: Plasma-derived Therapeutics*. Define therapeutic agents that are derived from plasma, such as Rh immune globulin, clotting factors, immunoglobulin, and albumin; discuss the evidence-based indications for their use.  

*Objective TM1.9: Pre-transfusion Procedures*. Discuss the key elements necessary to ensure safe and appropriate donation and transfusion of blood products.  

*Objective TM1.10: Blood Collection and Processing*. Discuss the basic blood collection procedure, including screening of blood donors, methods used, and potential complications.

### Topic: Hematology (H)

Every physician needs a thorough understanding of one of the most common tests ordered from the laboratory, the complete blood count (CBC). Differentiating tests needed for diagnosis and treatment of anemia and coagulation disorders is important for appropriate treatment and monitoring of these disorders.  

#### Learning Goal 1: Normal Coagulation. Retired.

Apply knowledge of biochemistry, pharmacology, and pathology to describe the basic cellular and molecular events associated with blood coagulation and explain laboratory tests for the diagnosis and management of coagulation disorders.  

*Objective H1.1: Platelet Aggregation After Injury*. Retired.  

*Objective H1.2: Platelet Inhibitors*. Moved to C3 H2.8.  

*Objective H1.3: Coagulation Cascade*. Retired. Use C1 HDTD2.1.  

*Objective H1.4: Anticoagulants*. Moved to C3 H2.9.  

#### Learning Goal 2: Diagnosis and Management of Coagulation Disorders

Apply knowledge of biochemistry, pharmacology, and pathology to describe the use of specific laboratory tests to diagnose and manage coagulation disorders.  

*Objective H2.1: Monitoring Anticoagulation Therapy*. Explain the selection of appropriate tests for identifying the cause(s) of bleeding and to monitor therapeutic anticoagulation.  

*Objective H2.2: Platelet Function Testing*. Explain platelet function testing and discuss how platelet function testing can be used to differentiate among disorders causing abnormal function of platelets.  

*Objective H2.3: Clotting Factor Deficiencies*. Describe how routine coagulation tests can be used to identify the likely deficiency of different clotting factor(s).  

*Objective H2.4: Evaluations of Coagulopathies*. Compare and contrast the roles of the following in evaluating coagulopathies: clinical history, prothrombin time (PT) test, partial thromboplastin time (PTT) test, D-dimer assay, platelet count, and platelet function tests.  

*Objective H2.5: Evaluation of the Bleeding Patient*. Describe how to evaluate a bleeding patient with a hemorrhagic disorder and explain how the history influences testing, including the uses and limitations of screening PT, PTT, and platelet counts.  

*Objective H2.6: Disseminated Intravascular Coagulation*. Retired. Use C3 H2.5.  

*Objective H2.7: Hereditary and Acquired Causes of Thrombosis*. Describe the major hereditary and acquired risk factors for thrombosis and how coagulation testing is used to confirm the diagnosis.  

*Objective H2.8: Platelet Inhibitors*. Explain the action and the clinical use of common platelet function inhibitor drugs, including, but not limited to, aspirin and clopidogrel.  

*Objective H2.9: Anticoagulants*. Explain the actions and clinical use of commonly used anticoagulants, including warfarin, the heparins, and the oral direct inhibitors of thrombin and factor Xa.  

#### Learning Goal 3: Mechanisms of Anemia

Apply knowledge of RBC structure/function and nutrient metabolism, the mechanisms of anemia, and the clinical and pathological features of common causes of anemia to develop an appropriate differential diagnosis.  

*Objective H3.1: RBC Function*. Summarize laboratory testing for key cellular structures and functions of the RBC.  

*Objective H3.2: Nutrients Required for Erythropoiesis*. Discuss the laboratory testing for specific nutrients, including iron and vitamins, needed for erythropoiesis.  

Objective H3.3: Blood Loss. Retired. Use C2 HRC1.8.  

#### Learning Goal 4: Diagnosis of the Anemic Patient

Apply knowledge of RBC structure/function and nutrient metabolism, the mechanisms of anemia, and the clinical and pathological features of common causes of anemia to develop an appropriate differential diagnosis of anemia.  

*Objective H4.1: Classification and Diagnosis of Anemia*. Discuss how anemia is classified; what tests are used in classification, and how follow-up testing is used to narrow differential diagnosis in different types of anemia.  

*Objective H4.2: Interpreting the CBC*. Explain the contribution of each of the measurements of the CBC, how they are derived, and how they can help diagnose blood cell disorders using specific examples.  

*Objective H4.3: Peripheral Smear Evaluation in Anemia*. Discuss the RBC and WBC morphology on a peripheral smear to develop a differential diagnosis for a patient with anemia.  

*Objective H4.4: Inherited Anemia*. Retired. Use C2-specific anemia Learning Objectives.  

*Objective H4.5: Acquired Anemia*. Retired. Use C2 HRC1.5.  

*Objective H4.6: Treatment of Anemia*. Retired.  

*Objective H4.7: Lab diagnosis*. Retired. Use C3 H4.1.  

#### Learning Goal 5: Diagnosis of White Cell Disorders

Apply knowledge of WBC structure, function, and development for patients with white cell disorders.  

*Objective H5.1: WBC Count and Differential*. Describe, with examples, how abnormalities of WBC count and WBC differential count can be used along with patient history to direct laboratory testing in the workup of a patient.  

*Objective H5.2: Lymphocyte Subsets*. Describe the subsets of lymphocytes that can be found in the peripheral blood and discuss how they are measured and how they may be altered in disease.  

*Objective H5.3: Neutrophils*. Discuss genetic factors that produce quantitative and qualitative abnormalities of neutrophils and how laboratory testing can diagnose diseases with these abnormalities.  

*Objective H5.4: Flow Cytometry*. Outline the principles of flow cytometry and describe, with examples, how it may be used to diagnose and monitor neoplastic and non-neoplastic WBC disorders.

### Topic: Microbiology (M)

Infectious diseases are extremely common, and every physician needs to be able to correlate clinical findings with the appropriate testing needed. Some infectious disorders will require immediate organism identification and susceptibility to pharmacotherapy, and understanding principles underlying the different types of microorganisms and their identification is essential. Many newer techniques have been recently implemented, including molecular techniques, that allow more definitive identification and specialized treatment for infectious organisms.  

#### Learning Goal 1: Pathogenesis, Diagnosis, and Treatment of Infectious Disease

Apply knowledge of infectious organisms to explain the pathogenesis of disease and clinical syndromes, appropriate collection of patient samples, organism identification and classification, antibiotic choice, and selection of medical/surgical interventions.  

*Objective M1.1: Preanalytic Factors in Microbiology*. Explain the types of preanalytical variables that affect diagnostic accuracy, including site and sterile/non-sterile specimens, and discuss factors that affect length of turn-around time for microbiological workups.  

*Objective M1.2: Gram Stain*. Moved to C3 M2.14.  

*Objective M1.3: Organism Identification*. Give examples of the types of testing performed in microbiology including cultures and their optimal usage to identify an infectious disease.  

*Objective M1.4: Coordination of Treatment*. Retired.  

*Objective M1.5: Rapid Testing in Microbiology*. Describe the uses and limitations of POC testing for infectious diseases from various body sites.  

*Objective M1.6 (Formerly C3 M2.9): Molecular Testing in Microbiology*. List examples of molecular tests that are commonly used in clinical microbiology and explain how they have an important impact on clinical care.  

#### Learning Goal 2: Bacteriology and Antimicrobials

Integrate concepts of bacteriology, susceptibility testing, and antimicrobial agents to identify bacterial infections and guide treatment.  

*Objective M2.1: Mechanisms of Antibiosis*. Associate mechanisms of action with antimicrobial agents, including the following: disruption of cell wall synthesis, inhibition of protein synthesis, inhibition of DNA synthesis, and antimetabolites.  

*Objective M2.2: Antimicrobial Activity*. State the spectrum of activity for common antimicrobial agents.  

*Objective M2.3: Antibiotic Resistance*. Describe mechanisms of resistance found in common pathogens including the following: penicillinase and mecA in *Staphylococcus* subspecies, vanA and vanB in *Enterococcus* subspecies, and extended spectrum beta-lactamases and carbapenemases in Enterobacteriaceae.  

*Objective M2.4: Antimicrobial Susceptibility Testing*. Describe the standardized techniques used in antimicrobial susceptibility testing, why standardization is important, and the differences between a qualitative and quantitative result.  

*Objective M2.5: Genetics of Antibiotic Susceptibility*. Name the genetic element detected by surrogate testing for cefoxitin and oxacillin susceptibility and describe how the results for *Staphylococcus* subspecies are used to predict activity of other beta-lactam antibiotics.  

*Objective M2.6: Choice of Antibiotics*. Describe how the microbiology laboratory determines if an isolate from a blood culture is susceptible or resistant. Describe how the pharmacokinetic/pharmacodynamic models may influence a clinician's choice of antibiotics given the susceptibility of an organism using specific examples.  

*Objective M2.7: Antimicrobial Stewardship*. Outline the principles that guide an institution's reporting cascade for the following: FDA indications, Clinical and Laboratory Standards Institute guidance, site of infection, institution formulary, and antimicrobial stewardship.  

*Objective M2.8: Institutional Antibiogram*. Use an institutional antibiogram to prescribe therapy before susceptibility test results are available.  

*Objective M2.9: Molecular Testing in Microbiology*. Moved to C3 M1.6.  

*Objective M2.10: Mass Spectrometry in Microbiology*. Explain how the application of matrix-assisted laser desorption/ionization-time of flight mass spectrometry in the clinical microbiology laboratory can impact patient care.  

*Objective M2.11: Urine Studies for Cystitis*. Explain the role of urine studies, including culture, in selecting antimicrobial therapy for infectious cystitis.  

*Objective M2.12: Diagnosis of Urinary Tract Infections (UTI)*. Describe a testing strategy for a typical uncomplicated community acquired UTI versus a nosocomial UTI in a patient with a Foley catheter and list the key microbiological tests in the diagnosis of UTIs.  

*Objective M2.13: Diagnosis and Management of Syphilis*. Explain the role of Venereal Disease Research Laboratory/rapid plasma reagin (VDRL/RPR) and *Treponema*-specific tests in the diagnosis and management of syphilis.  

*Objective M2.14 (Formerly M1.2): Gram Stain*. Compare and contrast the interpretations of Gram stains for rapid diagnosis of causative bacterial agents from sterile and contaminated sites and discuss the clinical settings where recognition of bacteria is most meaningful.  

#### Learning Goal 3: Virology

Integrate concepts of virology with diagnostic techniques including culture, molecular, and antigen diagnostics to identify viral infections and guide treatment.  

*Objective M3.1: Hepatotropic Viruses*. Describe the laboratory findings that diagnose hepatitis and correlate with the different possible clinical outcomes for each of the major hepatotropic viruses.  

*Objective M3.2: Influenza*. Explain the diagnosis of influenza in terms of diagnostic tests used, major antigens present, and the implications of a major shift in these antigens.  

*Objective M3.3: Serology, Nucleic acid amplification testing (NAAT), and Culture*. Describe the role of serology, NAAT, and culture in the diagnosis of viral infections and discuss the advantages and disadvantages of each in terms of both accuracy and rapidity of diagnosis and with respect to evaluation in an epidemic or pandemic.  

*Objective M3.4: HIV Infection*. Explain the testing strategy used to diagnose HIV and the role of viral load and CD4 count in monitoring HIV infection.  

*Objective M3.5: Response to HIV Treatment*. Describe the tests available to examine the response of an HIV virus to therapeutic agents, explaining how each test works.  

*Objective M3.6: Other Viral Infections*. Describe the testing strategy for other viral infections, including SARS viruses, respiratory syncytial virus, and congenitally acquired viruses.  

#### Learning Goal 4: Mycobacteria

Integrate concepts of mycobacteriology with diagnostic techniques, including culture, molecular, and antigen diagnostics, to identify mycobacterial infections and guide treatment.  

*Objective M4.1: Identification of Mycobacteria*. Describe the diagnostic tests available for the identification of mycobacteria, including culture methods and new molecular tests.  

*Objective M4.2: Antimycobacterial Susceptibility*. Compare and contrast the methods, culture, and molecular tests used to identify mycobacterial drug susceptibility and the time required for results by each method.  

#### Learning Goal 5: Mycology

Integrate concepts of mycology with diagnostic techniques, including culture, molecular, and antigen diagnostics to identify fungal infections and guide treatment.  

*Objective M5.1: Types of Fungi and Yeast*. Differentiate among filamentous fungi, dimorphic fungi, and yeast and describe the diagnostic approaches for each type.  

*Objective M5.2: Sensitivity Testing for Yeast*. Define sensitivity testing and describe its role and use in the management of yeast infections.  

*Objective M5.3: Special Testing for Fungi and Pneumocystis*. Explain the basis for the galactomannan and beta-glucan tests and how they are utilized to detect fungi and *Pneumocystis*.  

#### Learning Goal 6: Parasitology

Integrate concepts of parasitology with diagnostic techniques, including culture, molecular, and antigen diagnostics, to identify parasitic infections and guide treatment.  

*Objective M6.1: Metazoan and Protozoan Parasites*. Compare and contrast metazoan and protozoan parasites and the diagnostic approaches for each.  

*Objective M6.2: Stool Testing for Parasites*. Explain the role of stool samples, including number examined, role of microscopy, and detection, in the diagnosis of parasitic disease.  

*Objective M6.3: Serologic Testing for Parasites*. Summarize the role of serology and serological tests to diagnose toxoplasmosis and assess the risk of transmission during pregnancy.  

*Objective M6.4: Malaria and Babesiosis*. Contrast *Plasmodium falciparum* with other malaria species and babesiosis on a blood smear and explain the role of thick and thin smears in the diagnosis and management of malaria.  

*Objective M6.5: Rapid Testing for Malaria*. Name the rapid tests that do not require blood smears to identify malaria and explain how these tests work.

### Topic: Chemistry (CHEM)

Every physician needs to be able to differentiate among multiple different chemical tests in order to narrow differential diagnoses, confirm a diagnosis, or to follow disease progression. To treat patients appropriately, physicians need to understand the appropriate use of major clinical chemistry tests, the limitations of their use, and the pathophysiologic changes that produce alterations in their values.  

#### Learning Goal 1: Pathogenesis, Diagnosis, and Treatment of Common Disorders

Apply knowledge of clinical chemistry and physiology to discuss the role of chemistry testing in the laboratory in the diagnosis and management of common disorders and to describe how the pathophysiology of disease is reflected in abnormalities in results of chemistry tests.  

*Objective CHEM1.1: Thyroid Function Tests*. Discuss the efficient use of laboratory tests to identify and manage patients with thyroid disorders, to define the pathogenesis of these disorders, and to help distinguish among diseases in the differential diagnosis.  

*Objective CHEM1.2: Cardiac Disorders*. Discuss the efficient use of laboratory tests to identify and manage patients with cardiac disorders, to define the pathogenesis of these disorders, and to help distinguish among diseases in the differential diagnosis.  

*Objective CHEM1.3: Endocrine Disorders*. Discuss the efficient use of laboratory tests to identify and manage patients with endocrine disorders, to define the pathogenesis of these disorders, and to help distinguish among diseases in the differential diagnosis.  

*Objective CHEM1.4: Liver Disorders (Gastrointestinal Disease is moved to C3 CHEM1.9)*. Discuss the efficient use of laboratory tests to identify and manage patients with liver disorders, to define the pathogenesis of these disorders, and to help distinguish among diseases in the differential diagnosis.  

*Objective CHEM1.5: Renal Disorders*. Discuss the efficient use of laboratory tests to identify and manage patients with renal disorders, to define the pathogenesis of these disorders, and to help distinguish among diseases in the differential diagnosis.  

*Objective CHEM1.6: Lung Disease*. Discuss the efficient use of laboratory tests to identify and manage patients with lung disease, to define the pathogenesis of these disorders, and to help distinguish among diseases in a differential diagnosis.  

*Objective CHEM1.7: Toxicology*. Outline the value of testing for drugs and toxins, accounting for the routes of administration, distribution, and metabolism of the agent of interest and including the specimen source, the analytes to be detected given the medical questions, and the timing constraints for specimen collection.  

*Objective CHEM1.8: Cancer Diagnostics*. Discuss the appropriate tests for specific cancer diagnostics, including tumor markers and serum monoclonal protein analysis.  

*Objective CHEM1.9: Gastrointestinal Disorders (Formerly C3 CHEM1.4)*. Discuss the efficient use of laboratory tests to identify and manage patients with gastrointestinal disorders, to define the pathogenesis of these disorders, and to help distinguish among diseases in the differential diagnosis.  

*Objective CHEM 1.10: Muscle, Nerve, and Bone Disorders*. Discuss the efficient use of laboratory tests to identify and manage patients with muscle, bone, and nerve disorders; to define the pathogenesis of these disorders; and to help distinguish among diseases in the differential diagnosis.  

*Objective CHEM 1.11: Prenatal Disease*. Discuss the efficient use of clinical chemistry tests to identify and manage patients with prenatal disease, to define the pathogenesis of these disorders, and to help distinguish among diseases in the differential diagnosis.  

*Objective CHEM1.12: Diabetes Testing*. Discuss the efficient use of laboratory tests to diagnose and manage patients with diabetes mellitus, to define the pathogenesis of this disorder, to determine risk of development, and to monitor the disease process.  

*Objective CHEM 1.13: Therapeutic Drug Monitoring*. Outline the value of testing for therapeutic drugs, accounting for the routes of administration, distribution, and metabolism of the agent of interest and including the timing constraints for specimen collection.  

#### Learning Goal 2: Multisystem Panel Testing

Apply knowledge of chemistry and physiology to discuss the utility of panel testing in the laboratory in the diagnosis and management of common disorders and to describe how the pathophysiology of disease is reflected in abnormal results of chemistry tests.  

*Objective CHEM 2.1: Basic and Comprehensive Metabolic Panel*. Outline the components of the basic and comprehensive metabolic panels and describe their advantages and disadvantages as screening tests.  

*Objective CHEM 2.2: Lipid Panel*. Outline the components of the lipid panel and identify and describe how this test is used to diagnose and manage patients with lipid disorders.  

#### Learning Goal 3: Urinalysis and Body Fluids

Apply knowledge of normal chemical and cellular contents of urine, cerebrospinal fluid, and body cavity fluids in the diagnosis and management of common disorders and describe how the pathophysiology of disease is reflected in abnormal results of fluid tests.  

*Objective CHEM 3.1: Urinalysis*. Discuss the advantages and disadvantages of routine urinalysis and describe how particular abnormalities can help in the diagnosis and/or monitoring of specific diseases.  

*Objective CHEM 3.2: Body Fluid Testing*. Discuss the role of examination of effusions in helping to elucidate their causes.  

*Objective CHEM 3.3: Cerebral Spinal Fluid (CSF) Testing*. Outline the components of spinal fluid testing and describe how this test is used to diagnose and manage patients with CSF disorders.  

*Objective CHEM 3.4: Blood Gas Analysis*. Discuss the components of a blood gas panel and describe how these tests are used to manage patients, particularly in the intensive care unit or in the surgical setting.

### Topic: Immunology (IMM)

Every physician needs an understanding of specific laboratory tests to differentiate between inflammatory and immune-mediated diseases. Many newer techniques have been recently implemented that allow more definitive diagnosis and specialized treatment for these disorders.  

#### Learning Goal 1: Pathogenesis, Diagnosis, and Treatment of Immunologic Disorders

Apply knowledge of immunology, biochemistry, and pathology to describe the basic cellular and molecular events associated with immune system diseases of specific tissues and organ systems and the use of laboratory tests to diagnose and manage these diseases.  

*Objective IMM1.1: Markers of Inflammation*. Compare and contrast markers of inflammation in terms of the pathophysiologic basis and stages of the inflammatory response.  

*Objective IMM1.2: Immune Deficiencies and Allergen Testing*. Select and interpret appropriate tests for workup and interpretation of immunodeficiencies and allergy testing.  

*Objective IMM1.3: Serologic Testing for Infection*. Discuss, with examples, the application of serological testing in infectious diseases to establish immune status and diagnose infection.  

*Objective IMM1.4: Autoimmune Diseases*. Discuss the efficient use of laboratory tests to make a definitive diagnosis and manage autoimmune diseases.

### Topic: Molecular Pathology, Genomics, and Cytogenetics (GE)

Every physician needs an appreciation of the complex fields of molecular pathology, genomics, and cytogenetics, the ever-evolving molecular techniques that are essential for diagnosing diseases as accurately as possible, and the many diseases correlated with specific targeted immunotherapy or chemotherapy to maximize effectiveness of treatment, decrease side effects, and optimize patient survival.  

#### Learning Goal 1: Genes

Apply knowledge of genetics including the structure and organization of the human genome and regulation of gene expression, genetic variation, and inheritance patterns to basic disease processes.  

*Objective GE1.1: Mendelian Inheritance*. Retired. Use C1, GM1.2.  

*Objective GE1.2: Pedigrees*. Interpret a pedigree and discuss how this information can be applied for focused diagnostic testing.  

*Objective GE1.3: Inheritance Patterns*. Compare diagnostic testing of single-gene disorders to diseases with complex inheritance patterns and include the role of rare, high-risk variants and common, low-risk variants.  

*Objective GE1.4: Linkage Analysis*. Objective moved. Use C1 GM1.7.  

*Objective GE1.5: Population Genetics*. Objective moved. Use C1 GM1.8.  

*Objective GE1.6: Genetic Risk*. Objective moved. Use C1 GM1.9.  

*Objective GE1.7: Phenotypic Expression*. Objective moved. Use C1 GM1.10.  

*Objective GE1.8: Modifier Genes*. Objective moved. Use C1 GM1.11.  

*Objective GE1.9: Cytogenetics*. Define the following cytogenetic terms and nomenclature: karyotype, euploidy, aneuploidy, monosomy, trisomy, deletion, ring chromosome, inversion, isochromosome, translocation, balanced reciprocal translocation, and Robertsonian translocation.  

*Objective GE1.10: Mosaicism*. Objective moved. Use C1 GM1.13.  

#### Learning Goal 2: Chromosomal Disorders

Apply knowledge of genetics to explain the molecular basis of single-gene and non-neoplastic chromosomal disorders.  

*Objective GE2.1: Testing for Genetic Disorders*. Describe, with disease examples, how laboratory tests are used to diagnose the following specific genetic disorders: Mendelian disorders, autosomal disorders (dominant and recessive), X-linked disorders, chromosomal disorders, and disorders of nonclassic inheritance.  

#### Learning Goal 3: Genetic Basis of Neoplasia

Apply knowledge of genetics to explain the genetic basis for neoplasia and the role of genetic testing in diagnosis and treatment of diseases.  

*Objective GE3.1: Genetic Predisposition to Neoplasia*. Retired. Use C1 N1.1.  

*Objective GE3.2: Genetic Mechanisms of Neoplasia*. Retired. Use C1 N1.1.  

*Objective GE3.3: Molecular Testing in Oncology*. Explain the application of molecular testing for diagnosis, prognostication, and therapeutic follow-up of oncologic diseases.  

*Objective GE3.4: PCR Testing*. Discuss the advantages and limitations of PCR testing in oncology.  

*Objective GE3.5: Genome Testing*. Compare gene panel sequencing, whole exome sequencing, whole genome sequencing, and transcriptome sequencing with respect to the information gained from each, cost, and turnaround time and discuss clinical situations in which each might be appropriate.  

*Objective GE3.6: Chromosomal Alterations*. Compare and contrast techniques used to identify structural or numerical alterations in chromosomes and discuss how these can be used in the diagnosis and monitoring of patients with neoplasia.  

#### Learning Goal 4: Reproductive Genetics

Apply knowledge of genetics to explain the role of reproductive genetics and population screening.  

*Objective GE4.1: Carrier Testing*. Describe the role of preconception and prenatal carrier testing for genetic disorders depending upon family history and ethnic background.  

*Objective GE4.2: Newborn Screening*. Describe the rationale for newborn screening for genetic diseases and explain the difference between screening and diagnostic testing.  

#### Learning Goal 5: Diagnosis, Treatment, and Counseling

Apply knowledge of genetics to explain the role of genetic testing in diagnosis and treatment of diseases and in counseling of patients and families.  

*Objective GE5.1: Treatment Mechanisms*. Retired.  

*Objective GE5.2: Genetic Variation in Response to Treatment*. Discuss, with examples, how laboratory testing is used to identify genetic polymorphisms that can predict efficacy of medications and risk for adverse effects.  

*Objective GE5.3: Factors to Prevent Disease in the Genetically Predisposed*. Retired. Please use specific learning objectives from C1 Environmental Mechanisms.  

*Objective GE5.4: Genetic Counseling*. Discuss, with examples, how collaborations between genetic counselors and the laboratory optimize laboratory genetic testing for efficient and cost-effective patient care.  

*Objective GE5.5: Inherited Diseases*. Discuss the approach used for establishing the diagnosis for inherited diseases, directing therapy, and optimizing cost effective care.

### Topic: Autopsy (AU)

Autopsy is a division of anatomic pathology that encompasses the examination of a deceased person, either for medical or legal reasons. Understanding the value of the autopsy, both for scientific investigation of potential inherited disorders and for understanding the diseases that led to a patient's demise, will allow physicians to appropriately discuss this end-of-life medical evaluation.  

#### Learning Goal 1: Value of the Autopsy and Obtaining Consent

Apply knowledge of clinical medicine, quality management, and medicolegal issues to discuss the value of the autopsy and procedures for obtaining permission.  

*Objective AU1.1: Value of the Autopsy*. Provide examples demonstrating the value of the autopsy toward improvement in clinical diagnosis and management, quality control, medical education, research, and elucidation of “new” diseases.  

*Objective AU1.2: Consent for Autopsy*. Discuss the general process to identify the legal next of kin or individual authorized to consent for an autopsy.  

*Objective AU1.3: Obtaining Consent from the Family*. Describe how to approach a family to request consent for an autopsy, including a discussion of the autopsy procedure in language that the patient's family can understand.  

*Objective AU1.4: Professionalism in the Autopsy*. Discuss the psychosocial–emotional aspects of the autopsy experience, including its role in closure, and the importance of communication and professionalism among the healthcare team.  

*Objective AU1.5: Anatomic Findings in Autopsy*. Describe the role of the autopsy in identifying dysmorphology and anatomic abnormalities to help diagnose and classify congenital disorders.  

#### Learning Goal 2: Death Certificate

Apply knowledge of quality management to discuss the utility of death certificates and proper approaches for completing them.  

*Objective AU2.1: Public Health*. Describe the importance of death certificates for tracking and analysis of public health trends.  

*Objective AU2.2: Components of the Death Certificate*. Discuss the key components of the death certificate and distinguish among the cause of death (immediate, intermediate, and underlying), contributing conditions, and manner of death.  

*Objective AU2.3: Medical Errors*. Explain how underutilization or overutilization of medical care and incorrect diagnoses, therapeutics, or informed consent can lead to medical errors and give examples of how an autopsy can identify errors, thereby improving health care and decision-making.  

#### Learning Goal 3: Forensic Autopsy

Apply knowledge of clinical medicine and postmortem examination to discuss the indications for medical examiner referral and special procedures in the forensic postmortem examination.  

*Objective AU3.1: Role of the Medical Examiner*. Define the role of a medical examiner in terms of public health and protection of legal rights and describe differences between a forensic and medical autopsy.  

*Objective AU3.2: Reportable Deaths*. Identify circumstances of death that need to be reported to the medical examiner/coroner.

### Topic: Surgical Pathology (SP)

Surgical pathology is the area of anatomic pathology where all tissues or hardware removed from a patient are evaluated. Specimens sent to surgical pathology may range from minute endoscopic biopsies to large limb resections. Special techniques are commonly used by pathologists in evaluating these specimens that allow for definitive diagnosis and the recommendation of appropriate treatment for both benign and malignant lesions.  

#### Learning Goal 1: Role in Diagnosis

Apply knowledge of clinical medicine and pathology to describe the role surgical pathology plays in diagnosis and treatment of benign and malignant disorders. Use specific examples from the most common diseases and forms of cancer.  

*Objective SP1.1: Specimen Types*. Distinguish biopsies and excisions, describe the circumstances in which each is appropriate, and discuss the different types of information obtained from each type.  

*Objective SP1.2: Differential Diagnosis*. List the major differential diagnoses for a surgical pathology specimen obtained from a lesion or mass in a particular location, and describe appropriate further studies to resolve the differential.  

*Objective SP1.3: Special Studies*. Describe how a pathologist uses fresh, fixed, and frozen tissue for histochemical and immunohistochemical stains and genetic testing to arrive at a diagnosis, provide prognostic information, and direct specific therapy.  

*Objective SP1.4: Staging*. Describe the information that the pathologist obtains from a resected tissue specimen, how this information is reported, how it is combined with clinical information to stage the tumor, and how staging information is used to guide treatment.  

*Objective SP1.5: Frozen Section*. Explain the use of frozen sections and how the pathologist works with the patient care team to optimize and prioritize diagnosis and patient management.  

*Objective SP1.6 (Formerly CI FECT2.7, FECT2.9, and FECT2.13): Special Stains for Infectious Organisms*. Discuss, with examples, the use of special stains to identify bacteria, fungi, viruses, and parasites in tissues.  

#### Learning Goal 2: Immune and Infectious Diseases

Apply knowledge of clinical medicine and pathology to describe the roles surgical pathology plays in diagnosis and treatment of inflammatory disease, in particular those with immune or infectious etiologies.  

*Objective SP2.1: Examples of Inflammatory Conditions*. Give examples of specific sites and diseases in which certain pathologic diagnoses of inflammatory and/or infectious conditions are critical to treatment and prognosis.  

*Learning Goal 3: Congenital Disorders—Retired. Use AU Learning Goal 1 Objectives.*

#### Learning Goal 4: Interpretation of Reports

Apply knowledge of clinical medicine and communication skills to interpret pathology reports and communicate the results to patients in the context of risk assessment and patient prognosis. Determine appropriate action, including additional testing and clinical evaluation.  

*Objective SP4.1: Explaining the Report to the Patient*. Describe the components of a surgical pathology report, emphasizing how reports are structured to ensure clear communication of diagnostic information to the ordering physician.  

#### Learning Goal 5: Diagnostic Approach to Leukemia and Lymphomas

Apply knowledge of pathology and the application of differential diagnoses to discuss the diagnostic approach to leukemia and lymphomas and describe the relative roles of ancillary laboratory studies in classification.  

*Objective SP5.1: Special Studies*. Describe the roles of immunohistochemistry, flow cytometry, cytogenetics, and molecular diagnostics in the diagnosis and classification of lymphoma and explain how, with examples, different techniques are most appropriate in diagnosis, staging, and management of disease.  

*Objective SP5.2: Use of Special Studies*. Explain how the work up of lymph nodes at the frozen section bench differs from routine frozen sections.  

*Objective SP5.3: Differential Diagnosis*. Retired. Use C3 SP1.2.  

*Objective SP5.4 Role of Morphology*. Discuss how routine morphologic examination of bone marrow and tissue biopsies can be used to direct selection of testing required for the diagnosis of leukemia and lymphoma.

### Topic: Cytopathology (CYP)

Cytopathology focuses on the individual cellular components of disease. Cytopathological examination is an essential tool for its wide-ranging reach in screening, diagnostics, prognostics, and prevention of advanced disease states. Furthermore, the minimally and noninvasive nature of ascertaining most cytological specimens allows for immediate access to viable cellular material for advanced testing, molecular, and biochemical analyses.  

#### Learning Goal 1: General Principles

Apply knowledge of general and systems pathology to understand the meaning and context of cytologic diagnoses.  

*Objective CYP1.1: Obtaining the Specimen*. Compare and contrast the basic methods to obtain cytologic material for diagnosis, describe the settings in which these can be used to diagnose benign and malignant conditions, and discuss the limitations of each.  

*Objective CYP1.2: Categorizing Diagnostic Certainty*. Compare and contrast the degree of diagnostic certainty applied to general categorization in cytologic diagnosis.  

*Objective CYP1.3: Identifying Infectious Diseases*. Describe, with examples, the uses and limitations of cytology in identifying common infectious diseases.  

*Objective CYP1.4: Use of Cytology for Staging of Neoplasms*. Describe how cytologic specimens can add valuable information for tumor staging.  

#### Learning Goal 2: Cytologic Diagnosis

Apply knowledge of clinical medicine, pathology, and healthcare delivery to describe the advantages cytopathologic examination offers over conventional pathologic tissue examination.  

*Objective CYP2.1: Screening*. Describe how cytopathology is used as a screening test and discuss its advantages and limitations.  

*Objective CYP2.2: Ancillary Testing*. Describe how ancillary testing such as flow cytometry, immunohistochemistry, and molecular diagnostic testing is used in conjunction with cytology examination.  

*Objective CYP2.3: Cervical Cancer Screening*. Describe how to utilize current algorithms for cervical screening and discuss how human papilloma virus testing is used for management.  

*Objective CYP2.4: Fine Needle Aspiration*. Discuss the advantages and limitations of fine needle aspiration.  

## Authors’ note

The opinions and assertions expressed herein are those of the authors and do not reflect the official policy or position of the Uniformed Services University of the Health Sciences or the Department of Defense.

## Funding

The authors disclosed receipt of the following financial support for the research, authorship, and/or publication of this article: The publication of the Pathology Competencies for Medical Education is supported by the Association of Pathology Chairs.

## Declaration of competing interest

The authors declared no potential conflicts of interest with respect to the research, authorship, and/or publication of this article.
